# Morphological Brain Networks of White Matter: Mapping, Evaluation, Characterization, and Application

**DOI:** 10.1002/advs.202400061

**Published:** 2024-07-15

**Authors:** Junle Li, Suhui Jin, Zhen Li, Xiangli Zeng, Yuping Yang, Zhenzhen Luo, Xiaoyu Xu, Zaixu Cui, Yaou Liu, Jinhui Wang

**Affiliations:** ^1^ Institute for Brain Research and Rehabilitation South China Normal University Guangzhou 510631 China; ^2^ State Key Laboratory of Cognitive Neuroscience and Learning Beijing Normal University Beijing 100875 China; ^3^ Chinese Institute for Brain Research Beijing 102206 China; ^4^ Department of Radiology Beijing Tiantan Hospital Beijing 100070 China; ^5^ Key Laboratory of Brain Cognition and Education Sciences Ministry of Education Guangzhou 510631 China; ^6^ Center for Studies of Psychological Application South China Normal University Guangzhou 510631 China; ^7^ Guangdong Key Laboratory of Mental Health and Cognitive Science South China Normal University Guangzhou 510631 China

**Keywords:** brain network, gene, magnetic resonance imaging, reliability, white matter

## Abstract

Although white matter (WM) accounts for nearly half of adult brain, its wiring diagram is largely unknown. Here, an approach is developed to construct WM networks by estimating interregional morphological similarity based on structural magnetic resonance imaging. It is found that morphological WM networks showed nontrivial topology, presented good‐to‐excellent test‐retest reliability, accounted for phenotypic interindividual differences in cognition, and are under genetic control. Through integration with multimodal and multiscale data, it is further showed that morphological WM networks are able to predict the patterns of hamodynamic coherence, metabolic synchronization, gene co‐expression, and chemoarchitectonic covariance, and associated with structural connectivity. Moreover, the prediction followed WM functional connectomic hierarchy for the hamodynamic coherence, is related to genes enriched in the forebrain neuron development and differentiation for the gene co‐expression, and is associated with serotonergic system‐related receptors and transporters for the chemoarchitectonic covariance. Finally, applying this approach to multiple sclerosis and neuromyelitis optica spectrum disorders, it is found that both diseases exhibited morphological dysconnectivity, which are correlated with clinical variables of patients and are able to diagnose and differentiate the diseases. Altogether, these findings indicate that morphological WM networks provide a reliable and biologically meaningful means to explore WM architecture in health and disease.

## Graphical abstract

This study proposes an approach to construct brain networks of white matter by estimating interregional morphological similarity based on structural magnetic resonance imaging. By combining multimodal and multiscale data from multiple datasets, morphological white matter networks are demonstrated to be test‐retest reliable, phenotypically related, genetically originated, functionally and structurally relevant, neurobiologically meaningful, and clinically valuable.

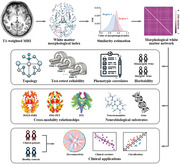

## 1 Introduction

The human brain operates essentially as an interconnected complex network in favor of behavior and cognition.^[^ ^1^ ^]^ This feature makes the brain particularly amenable to research from the perspective of modern network theory.^[^ ^2^ ^]^ To date, great progress has been made in the last decade in uncovering the organizational principles that govern the human brain networks and in revealing alterations of the human brain networks in development, aging, and diseases.^[^ ^3^ ^]^ However, current attention is mainly focused on the connectivity patterns between gray matter regions, which are typically thought to be responsible for brain function. In addition to gray matter, white matter (WM) is another important type of brain tissue, which accounts for nearly half of adult brain. However, despite being an important component of the brain, WM has rarely been investigated with respect to its wiring diagram.

Brain connectivity can be inferred in vivo from non‐invasive multimodal magnetic resonance imaging (MRI) techniques, such as functional connectivity estimated as temporal synchronization of neural activity typically recorded by functional MRI (fMRI) or structural connectivity reconstructed via fiber tractography from diffusion MRI. Using the functional connectivity approach, several pioneer studies have shown that neural activities in WM are organized as large‐scale functional networks with synchronous signal fluctuations in specific sets of WM tracts.^[^ ^4^ ^]^ Moreover, functional WM networks are found to exhibit non‐random topology, account for interindividual differences in general fluid intelligence, and be altered in brain disorders^[^ ^4^, ^5^ ^]^ With regard to the structural connectivity, a more recent study reconstructed whole‐brain WM fiber tracts, and found that the number of fiber tracts between WM regions were correlated with WM functional connectivity estimated from intracranial stereotactic‐electroencephalography and resting‐state fMRI.^[^ ^6^ ^]^ These findings collectively suggest that WM regions, like gray matter, also work together in a coordinated manner rather than in isolation. Thus, a comprehensive mapping and characterization of WM brain networks is crucial for fully understanding the whole‐brain network architecture.

Apart from the functional and structural connectivity, morphological similarity has become a growing focus of brain network studies over the past few years.^[^ ^7^ ^]^ Morphological similarity refers to statistical relationship between regions in local brain morphology, which can be quantified by various features from structural MRI, such as gray matter volume and cortical thickness. Morphological similarity provides an important complement to the functional and structural connectivity for studying brain networks, and has unique advantages given the high spatial resolution, high signal‐to‐noise ratio, stability over time, wide use, and easy access of structural MRI. Furthermore, evidence from gray matter studies has indicated that morphological similarity shows high test‐retest (TRT) reliability,^[^ ^8^ ^]^ is related to interindividual differences in behavior and cognition,^[^ ^9^ ^]^ has neurobiological underpinnings,^[^ ^9^, ^10^ ^]^ and is of clinical value in helping disease diagnosis.^[^ ^11^ ^]^ Despite these favorable characteristics, morphological similarity has rarely been used to characterize interregional relations in WM morphology.

In this study, we proposed an approach to construct WM networks by measuring the morphological similarity with two widely used WM morphological features: volume (reflecting a composite of thickness, area, and folding) and deformation (reflecting local tissue expansion or contraction). We first validated the approach by examining the topological organization of the constructed WM networks (hereafter referred to as morphological WM networks). Then, we evaluated both short‐term and long‐term TRT reliability of the morphological WM networks. Afterward, we examined the ability of the morphological WM networks to explain interindividual differences in a broad range of behavioral and cognitive measures, and evaluated the extent to which the morphological WM networks were under genetic control. After these analyses, we explored the cross‐modality relationship of morphological WM networks with hamodynamic coherence derived from resting‐state fMRI data, metabolic synchronization derived from resting‐state 18F‐fluorodeoxyglucose positron emission tomography (PET) data, and structural connectivity derived from diffusion MRI data. To better understand the morphological WM networks, we further linked them with brain‐wide transcriptional profiles and neurotransmitter distributions to analyze their genetic and chemoarchitectonic correlates, respectively. Finally, we applied the morphological WM networks to a multicentric dataset of multiple sclerosis (MS) and neuromyelitis optica spectrum disorders (NMOSD) to examine their clinical value in helping diagnose and differentiate the two diseases. Based on our findings, we argue that morphological WM networks provide a TRT reliable, phenotypically related, genetically originated, functionally and structurally relevant, neurobiologically meaningful, and clinically valuable approach for studies of human brain WM.

## 2 Results

### 2.1 General Description of Datasets, Analytical Methods, and Research Questions

In this study, we proposed an approach to construct morphological WM networks using structural MRI data. Briefly, for a structural MRI image, a voxel‐based morphometry method was first used to derive a WM volume map and deformation map. Then, these two maps were separately used to construct a morphological WM network (i.e., volume‐based network, VBN, and deformation‐based network, DBN) by estimating interregional morphological similarity among 48 WM regions of interest (ROIs)^[^ ^12^ ^]^ (Table S1, Supporting Information) via a Jensen‐Shannon divergence‐based similarity (JSDs) method.^[^ ^8e^ ^]^ For the constructed morphological WM networks, seven independent datasets were utilized to depict, evaluate, and characterize them from different perspectives, including organizational principle, TRT reliability, phenotypic association, heritability, functional and structural relevance, neurobiological substrate, and clinical value. Table S2 (Supporting Information) summarizes the demographic information for each dataset. Specifically, the Human Connectome Project (HCP) dataset (www.humanconnectome.org)^[^ ^13^ ^]^ was used to investigate the topological organization (unrelated participants), phenotypic association (unrelated participants), heritability (twin participants), and relationship with hamodynamic coherence and structural connectivity (unrelated participants) of morphological WM networks; the Hangzhou Normal University (HNU) dataset (https://doi.org/10.15387/fcp_indi.corr.hnu1)^[^ ^14^ ^]^ and South West University (SWU) dataset (https://doi.org/10.15387/fcp_indi.retro.slim)^[^ ^15^ ^]^ were used to examine the short‐term and long‐term TRT reliability of morphological WM networks, respectively; the Monash University (MU) dataset (https://openneuro.org/datasets/ds002898/versions/1.1.0)^[^ ^16^ ^]^ was used to explore the association of morphological WM networks with metabolic synchronization; the JuSpace dataset (https://github.com/juryxy/JuSpace)^[^ ^17^ ^]^ was used to construct a chemoarchitectonic WM network for analyzing molecular relevance of morphological WM networks; the Allen Human Brain Atlas (AHBA) dataset (http://human.brain‐map.org/)^[^ ^18^ ^]^ was used to construct a transcriptional WM network for examining genetic correlates of morphological WM networks; and the MS and NMOSD multicentric dataset^[^ ^19^ ^]^ was used to study clinical value of morphological WM networks. Structural MRI images in all the datasets underwent the same analytical pipeline to construct morphological WM networks unless stated otherwise.

### 2.2 Similarity Patterns of Morphological WM Networks (HCP Dataset, Unrelated Participants)

**Figure 1:**
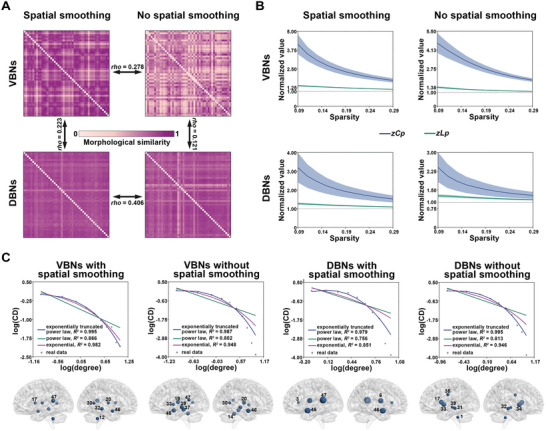
Topological descriptions of morphological WM networks. A) Mean interregional morphological similarity matrices and their spatial similarities across different analytical strategies. High morphological similarities were observed for most edges regardless of the choice of morphological index and whether spatial smoothing was performed. Spatial correlation analysis revealed significant but relatively low Spearman rank correlations in the group‐level mean morphological similarity matrix between different analytical strategies (*N* = 1128 edges; Spearman correlation, *P* < 0.05, FDR corrected). B) Global organization of morphological WM networks. Compared with matched random networks, the morphological WM networks exhibited higher clustering coefficient and approximately equal characteristic path length, indicative of small‐world organization. Data are presented as the mean ± standard deviation of 444 unrelated participants in the HCP dataset. C) Local organization of morphological WM networks. The degree distribution of the group‐level mean morphological WM networks was best fitted by an exponentially truncated power law model (top panel), indicative of the existence of highly connected brain regions (i.e., hubs; bottom panel). The numbers in the brain maps indicate the indices of the hubs in the WM atlas from Johns Hopkins University. See Table S2 (Supporting Information) for details. Dot sizes are proportional to regional values of nodal degree. VBNs, volume‐based networks; DBNs, deformation‐based networks; *zC_p_*, normalized clustering coefficient; *zL_p_*, normalized characteristic path length.

For each of the 444 unrelated healthy participants in the HCP dataset, we constructed two VBNs and two DBNs based on the smoothed (Gaussian kernel with 8‐mm full width at half maximum) and unsmoothed WM volume map and deformation map, respectively. **Figure** **1**A shows the group‐level mean morphological similarity matrices of the constructed morphological WM networks. In general, the interregional morphological similarities were high for most edges regardless of the choice of morphological index and whether spatial smoothing was performed. Nonetheless, specifically organized patterns were obvious. In particular, the mean interregional morphological similarity for homotopic edges linking geometrically corresponding regions between the two hemispheres was consistently found to be significantly higher than that for heterotopic edges linking non‐homotopic regions (independent‐sample *T* test; spatial smoothing: *T_859_* = 3.257, *P* = 0.001 for the VBNs and *T_859_* = 3.364, *P* < 0.001 for the DBNs; no spatial smoothing: *T_859_* = 1.984, *P* = 0.048 for the VBNs and *T_859_* = 1.784, *P* = 0.075 for the DBNs).

We further found that the group‐level mean morphological similarity matrices exhibited significant but relatively low Spearman rank correlations between the VBNs and DBNs (spatial smoothing: *rho* = 0.223, *P* < 0.001; no spatial smoothing: *rho* = 0.121, *P* = 0.003) and between spatially smoothed and unsmoothed data (VBNs: *rho* = 0.278, *P* < 0.001; DBNs: *rho* = 0.406, *P* < 0.001). Two‐way repeated ANOVA revealed that morphological index (*F_1,1127_* = 1118.672, *P* < 0.001) and spatial smoothing (*F_1,1127_* = 1773.517, *P* < 0.001) significantly affected interregional morphological similarity in an interactive manner (*F_1,1127_* = 1185.991, *P* < 0.001). Post hoc comparisons (paired‐sample *T* test) revealed that the DBNs had significantly higher morphological similarity than the VBNs no matter whether spatial smoothing was performed (spatial smoothing: *T_1127_* = 5.195, *P* < 0.001; no spatial smoothing: *T_1127_* = 42.597, *P* < 0.001), and spatial smoothing was associated with significantly higher morphological similarity for both the VBNs (*T_1127_* = 40.970, *P* < 0.001) and DBNs (*T_1127_* = 12.136, *P* < 0.001). These findings indicate distinct wiring patterns of morphological WM networks between the VBNs and DBNs and profound effects of spatial smoothing on morphological WM networks.

### 2.3 Topological Organization of Morphological WM Networks (HCP Dataset, Unrelated Participants)

For the morphological WM networks derived from the 444 unrelated healthy participants in the HCP dataset, we calculated a set of graph‐based network measures to study their topological organization, including small‐world organization, rich‐club architecture, degree distribution, and hubs.

#### 2.3.1 Small‐World Organization

Each morphological WM network exhibited typical small‐world organization regardless of the choice of morphological index and whether spatial smoothing was performed as characterized by the combination of normalized clustering coefficient > 1 and normalized characteristic path ≈1 (Figure 1B).

#### 2.3.2 Rich‐Club Architecture

Each morphological WM network exhibited a normalized rich‐club coefficient > 1 over a consecutive range of degree thresholds regardless of the choice of morphological index and whether spatial smoothing was performed, a typical feature of rich‐club organization.

#### 2.3.3 Hubs

The degree distribution of the group‐level mean morphological WM networks was best fitted by an exponentially truncated power law model regardless of the choice of morphological index and whether spatial smoothing was performed (Figure 1C, top). This form of degree distribution indicates the existence of highly connected regions (i.e., hubs) in the morphological WM networks, such as the right uncinated fasciculus and the left tapetum (Figure 1C, bottom).

### 2.4 TRT Reliability of Morphological WM Networks (HNU and SWU Datasets)

We utilized the intraclass correlation coefficient (*ICC*) to quantify the TRT reliability of morphological WM networks. Specifically, we calculated the *ICC* for the morphological similarity of each edge in the morphological WM networks derived from the HNU and SWU datasets. The HNU dataset was used to estimate short‐term TRT reliability, in which participants were scanned every three days over one month, whereas the SWU dataset was used to estimate long‐term TRT reliability, wherein participants were scanned three times (the average time intervals were 304.14, 515, and 817.87 days between the first and second scans, second and third scans, and first and third scans, respectively).

**Figure 2:**
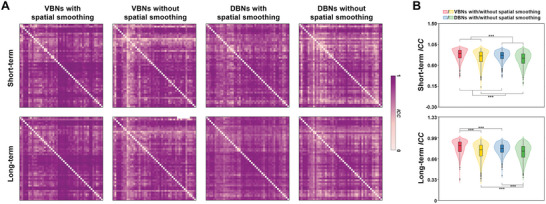
TRT reliability of morphological WM networks. A) Good‐to‐excellent short‐term (10 scans of 30 participants in the HNU dataset) and long‐term (3 scans of 120 participants in the SWU dataset) TRT reliabilities were observed for most edges in the morphological WM networks. B) Violin plots showing effects of different analytical strategies on the TRT reliabilities of morphological WM networks. Significant main effects of morphological index and spatial smoothing were observed on both the short‐term and long‐term TRT reliabilities of morphological WM networks (*N* = 1128 edges; two‐way repeated ANOVA, all *P* < 0.001). Post hoc comparisons revealed that the VBNs exhibited significantly higher TRT reliabilities than the DBNs and the implementation of spatial smoothing significantly increased the reliabilities of morphological WM networks (short‐term: *N* = 2256 edges; long‐term: *N* = 1128 edges; paired‐sample *T* test, *P* < 0.05, FDR corrected). Boxes in the plots indicate the median values with interquartile ranges. ***, *P* < 0.001; VBNs, volume‐based networks; DBNs, deformation‐based networks; *ICC*, intraclass correlation coefficient.

Good‐to‐excellent short‐term and long‐term TRT reliabilities (*ICC* > 0.6) were observed for most edges in the morphological WM networks regardless of the choice of morphological index and whether spatial smoothing was performed (**Figure** **2**A). For example, the *ICC* values (mean ± standard deviation) of the VBNs were 0.832 ± 0.115 and 0.844 ± 0.103 for short‐term and long‐term TRT reliability, respectively, when spatial smoothing was performed. Two‐way repeated ANOVA revealed significant main effects of morphological index (*F_1,1127_* = 120.381, *P* < 0.001) and spatial smoothing (*F_1,1127_* = 610.157, *P* < 0.001) on the short‐term TRT reliability of morphological WM networks. Post hoc comparisons (paired‐sample *T* test) revealed that the VBNs exhibited significantly higher short‐term reliability than the DBNs (*T_2255_* = 12.182, *P* < 0.001) and the implementation of spatial smoothing significantly increased the short‐term reliability of morphological WM networks (*T_2255_* = 26.276, *P* < 0.001). For the long‐term TRT reliability, morphological index (*F_1,1127_* = 91.805, *P* < 0.001) and spatial smoothing (*F_1,1127_* = 722.086, *P* < 0.001) significantly affected morphological WM networks in an interactive manner (*F_1,1127_* = 8.626, *P* = 0.003). Post hoc comparisons (paired‐sample *T* test) revealed that the VBNs exhibited significantly higher long‐term reliability than the DBNs no matter whether spatial smoothing was performed (spatial smoothing: *T_1127_* = 10.442, *P* < 0.001; no spatial smoothing: *T_1127_* = 5.829, *P* < 0.001) and the implementation of spatial smoothing significantly increased the long‐term reliability of both the VBNs (*T_1127_* = 21.702, *P* < 0.001) and DBNs (*T_1127_* = 20.991, *P* < 0.001) (Figure 2B).

Based on these findings, only morphological WM networks derived from spatially smoothed data were used for the following analyses.

### 2.5 The Relationship between Morphological WM Networks and Behavior and Cognition (HCP Dataset, Unrelated Participants)

The HCP dataset included a broad range of behavioral and cognitive measures, which allowed examining phenotypic correlates of morphological WM networks. Specifically, we used the partial least‐squares (PLS) regression to examine the ability of morphological WM networks to explain interindividual variance in six behavioral and cognitive domains, including Alertness, Cognition, Emotion, Motor, Personality, and Sensory. Only the first component of the PLS model (i.e., PLS1) was examined, which was the linear combination of interregional morphological similarities that exhibited the strongest correlation with the behavioral and cognitive data. In addition, we employed the brain bias set (BBS) modeling method^[^ ^20^ ^]^ to examine the ability of morphological WM networks to predict individual scores in the six behavioral and cognitive domains.

**Figure 3:**
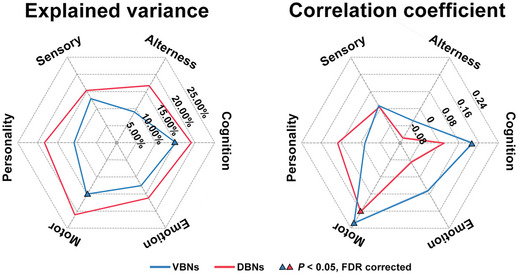
Behavioral and cognitive association of morphological WM networks. The VBNs explained significant proportions of interindividual variance in the Cognition and Motor domains (partial least‐squares regression; left panel) and the VBNs and DBNs significantly predicted individual scores of the Cognition and Motor domains and the Motor domain, respectively (brain bias set modeling method; right panel) (*N* = 444 unrelated participants in the HCP dataset; *P* < 0.05, FDR corrected). VBNs, volume‐based networks; DBNs, deformation‐based networks; FDR, false discovery rate.

We found that the PLS1 scores derived from the VBNs explained significant proportions of interindividual variance in the Cognition (15.1%, *P* = 0.006) and Motor (15.1%, *P* = 0.006) domains (**Figure** **3**, left), whereas no significant results were found for the DBNs. For the prediction analyses, we found that the VBNs predicted individual scores of the Cognition (mean *r* = 0.134, *P* = 0.013) and Motor (mean *r* = 0.215, *P* < 0.001) domains and the DBNs predicted individual scores of the Motor domain (mean *r* = 0.161, *P* = 0.005) (Figure 3, right).

### 2.6 Heritability of Morphological WM Networks (HCP Dataset, Twin Participants)

To investigate the extent to which morphological WM networks were genetically controlled, a genetic ACE model was used to quantify the heritability for the morphological similarity of each edge in the morphological WM networks derived from the 217 pairs of monozygotic (MZ) and dizygotic (DZ) twins in HCP dataset.

**Figure 4:**
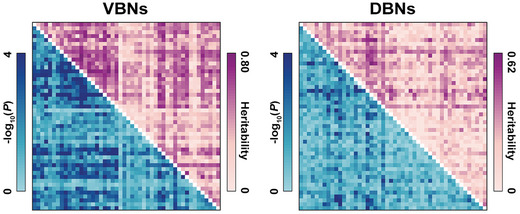
Heritability of morphological WM networks. In general, morphological WM networks exhibited low‐to‐moderate heritability (*N* = 217 pairs of monozygotic and dizygotic twins in the HCP dataset; genetic ACE model). Moreover, the VBNs exhibited significantly higher heritability than the DBNs (*N* = 1128 edges; paired‐sample *T* test, *P* < 0.001). VBNs, volume‐based networks; DBNs, deformation‐based networks.

In general, low‐to‐moderate heritability was observed for both the VBNs (0.382 ± 0.195) and DBNs (0.207 ± 0.139). Compared with the DBNs, the VBNs were associated with significantly higher heritability (paired‐sample *T* test, *T_1127_* = 29.337, *P* < 0.001). Moreover, the heritability showed a spatially heterogeneous distribution with a specific set of edges under strong genetic control (VBNs: 586 edges, 0.522 ± 0.125; DBNs: 137 edges, 0.359 ± 0.112) [*P* < 0.05, false discovery rate (FDR) corrected] (**Figure** **4**). Interestingly, we found that the heritability of edges in the VBNs exhibited significant positive Spearman correlations with the edges’ contributions to explaining interindividual variance in the Motor (*rho* = 0.273, *P* < 0.001) and Cognition (*rho* = 0.126, *P* = 0.024) domains and contributions to predicting individual scores in the Cognition domain (*rho* = 0.109, *P* = 0.021).

### 2.7 Relationship between Morphological WM Networks and Hamodynamic Coherence (HCP Dataset, Unrelated Participants)

To examine functional correlates of morphological WM networks, we constructed functional WM networks by estimating interregional hamodynamic coherence among the 48 WM ROIs based on resting‐state fMRI data. Then, the communication model, a multiple linear regression model based on simple dynamical processes,^[^ ^21^ ^]^ was used to predict the group‐level mean hamodynamic coherence network with the group‐level mean morphological WM network and the group‐level mean networks of communicational organization (shortest path length and communicability) derived from the morphological WM networks. These analyses were performed for the unrelated healthy participants in the HCP dataset.

**Figure 5:**
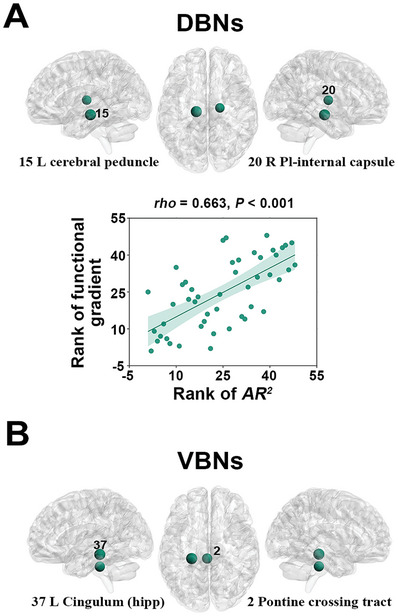
Relationship between morphological WM networks and hamodynamic coherence and metabolic synchronization networks. A) The DBNs significantly predicted the hamodynamic coherence profiles of the left cerebral peduncle and right posterior limb of internal capsule (*N* = 47 edges; multiple linear regression model, *P* < 0.05, false discovery rate corrected; top panel). Moreover, regional *AR^2^* values exhibited a significant positive correlation with the first gradient derived from group‐level mean WM hamodynamic coherence network (*N* = 48 regions; Spearman correlation, *P* < 0.001; bottom panel). B) The VBNs significantly predicted the metabolic synchronization profiles of the pontine crossing tract and left cingulum hippocampus (*N* = 47 edges; multiple linear regression model, *P* < 0.05, false discovery rate corrected). The numbers in the brain maps indicate the indices of the identified regions in the WM atlas from Johns Hopkins University. Dot sizes are proportional to regional *AR^2^* values. *AR^2^*, adjusted R‐squared; VBNs, volume‐based networks; DBNs, deformation‐based networks.

At the whole‐brain level, both the VBNs [adjusted R‐squared (*AR^2^*) = 0.034, *P* = 0.004] and DBNs (*AR^2^* = 0.022, *P* = 0.017) significantly predicted the pattern of the hamodynamic coherence network. For node‐wise multilinear predictions, no significant predictions were observed for the VBNs (*AR^2^* = −0.061–0.192; *P* > 0.05, FDR corrected). For the DBNs (*AR^2^* = −0.055–0.417), significant predictions were observed for the left cerebral peduncle (*AR^2^* = 0.417, *P* < 0.001) and the right posterior limb of internal capsule (*AR^2^* = 0.362, *P* < 0.001) (**Figure** **5**A, top).

We further examined whether the nodal *AR^2^* values for the DBNs followed the hierarchical organization of WM hamodynamic coherence networks. The hierarchical organization was obtained using the BrainSpace toolbox,^[^ ^22^ ^]^ which yielded gradient maps that accounted for primary variations in the distribution of hamodynamic coherence across the WM ROIs. We found that the first gradient accounted for 30.5% variance in the distribution of hamodynamic coherence across the WM ROIs. This gradient was characterized by gradual reduction of hamodynamic coherence variance along an axis from inferior to superior WM regions (Figure S1, Supporting Information). We found that the nodal *AR^2^* values exhibited a significant positive correlation with the gradient for the DBNs (*rho* = 0.663, *P* < 0.001) (Figure 5A, bottom).

### 2.8 Relationship between Morphological WM Networks and Metabolic Synchronization (MU Dataset)

To further examine functional correlates of morphological WM networks, we constructed functional WM networks by estimating interregional metabolic synchronization among the 48 WM ROIs based on resting‐state PET data. Subsequently, the communication model was used to examine the ability of the group‐level mean morphological WM network to predict the group‐level mean metabolic synchronization network. These analyses were performed for the 26 healthy participants in MU dataset.

At the whole‐brain level, the VBNs (*AR^2^* = 0.012, *P* = 0.017) but not DBNs (*AR^2^* = 0.005, *P* = 0.411) significantly predicted the pattern of the metabolic synchronization network. For node‐wise multilinear predictions, no significant predictions were observed for the DBNs (*AR^2^* = −0.067–0.251; *P* > 0.05, FDR corrected). For the VBNs (*AR^2^* = −0.067–0.282), significant predictions were found for the pontine crossing tract (*AR^2^* = 0.260, *P* < 0.001) and the left cingulum hippocampus (*AR^2^* = 0.282, *P* < 0.001) (Figure 5B).

We further linked the nodal *AR^2^* values for the VBNs with regional static metabolic values, and found no significant correlation (*P* > 0.05).

### 2.9 Relationship between Morphological WM Networks and Structural Connectivity (HCP Dataset, Unrelated Participants)

To examine structural correlates of morphological WM networks, we constructed structural WM networks by estimating interregional structural connectivity among the 48 WM ROIs based on diffusion MRI data. Again, the communication model was used to examine the ability of the group‐level mean morphological WM network to predict the group‐level mean structural connectivity network. These analyses were performed for the unrelated healthy participants in the HCP dataset.

At the whole‐brain level, neither the VBNs (*AR^2^* = 0.004, *P* = 0.342) nor DBNs (*AR^2^* = 0.016, *P* = 0.132) significantly predicted the pattern of the structural connectivity network. This was also the case for node‐wise multilinear predictions (VBNs: *AR^2^* = −0.069–0.273; DBNs: *AR^2^* = −0.058–0.343) (*P* > 0.05, FDR corrected).

Considering that structural WM networks contained many zeros, which may result in an underestimation of the relationship between morphological and structural WM networks for the multiple linear regression model, we further compared the morphological similarity between region pairs with and without structural connectivity (paired‐sample *T* test). Compared with region pairs without structural connectivity, structurally connected region pairs exhibited significantly higher morphological similarity for the VBNs (*T_412_* = 18.716, *P* < 0.001) but lower morphological similarity for the DBNs (*T_412_* = −8.147, *P* < 0.001).

### 2.10 Relationship between Morphological WM Networks and Chemoarchitectonic Covariance (JuSpace Dataset and HCP Dataset, Unrelated Participants)

Mounting evidence indicates the existence of multiple neurotransmitters in WM tracts, such as serotonergic, glutamatergic, dopaminergic, GABAergic, purinergic, adrenergic, and cholinergic signaling.^[^ ^23^ ^]^ To understand potential neurobiological substrate of morphological WM networks, we constructed a chemoarchitectonic WM network by estimating the chemoarchitectonic covariance among the 48 WM ROIs based on 26 neurotransmitter distribution maps included in the JuSpace dataset. The communication model was subsequently used to predict the chemoarchitectonic covariance network with the group‐level mean morphological WM network derived from the unrelated healthy participants in the HCP dataset.

**Figure 6:**
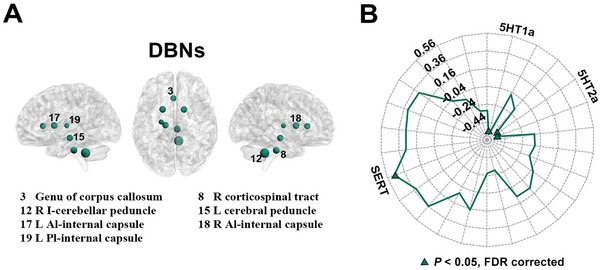
Relationship between morphological WM networks and chemoarchitectonic covariance network. A) The DBNs significantly predicted the chemoarchitectonic covariance profiles of the genu of corpus callosum, left cerebral peduncle, left anterior limb of internal capsule, left posterior limb of internal capsule, right corticospinal tract, right inferior cerebellar peduncle and right anterior limb of internal capsule (*N* = 47 edges; multiple linear regression model, *P* < 0.05, FDR corrected). The numbers in the brain maps indicate the indices of the identified regions in the WM atlas from Johns Hopkins University. Dot sizes are proportional to regional *AR^2^* values. B) Regional *AR^2^* values exhibited significant correlations with regional neurotransmitter density of the SERT with the ^11^C‐MADAM tracer, 5HT1a with the ^11^C‐CUMI tracer, and 5HT2a with the ^18^F‐ALTANSERIN tracer and ^11^C‐CIMBI tracer (*N* = 48 regions; Spearman correlation, *P* < 0.05, FDR corrected). *AR^2^*, adjusted R‐squared; DBNs, deformation‐based networks; FDR, false discovery rate.

At the whole‐brain level, the DBNs (*AR*  = 0.056, *P* = 0.007) but not VBNs (*AR^2^* = 0.022, *P* = 0.145) significantly predicted the pattern of the chemoarchitectonic covariance network. For node‐wise multilinear predictions, no significant predictions were observed for the VBNs (*AR^2^* = −0.067–0.321; *P* > 0.05, FDR corrected). For the DBNs (*AR^2^* = −0.067–0.503), significant predictions were observed for the genu of corpus callosum (*AR^2^* = 0.296, *P* < 0.001), the left cerebral peduncle (*AR^2^* = 0.318, *P* < 0.001), the left anterior limb of internal capsule (*AR^2^* = 0.333, *P* < 0.001), the left posterior limb of internal capsule (*AR^2^* = 0.227, *P* = 0.003), the right corticospinal tract (*AR^2^* = 0.344, *P* < 0.001), the right inferior cerebellar peduncle (*AR^2^* = 0.503, *P* < 0.001), and the right anterior limb of internal capsule (*AR^2^* = 0.322, *P* < 0.001) (**Figure** **6**A).

We further examined the association of the nodal *AR^2^* values for the DBNs with regional density of each neurotransmitter. A significant positive correlation was observed for the SERT with ^11^C‐MADAM tracer (*rho* = 0.476, *P* = 0.002) and significant negative correlations were observed for the 5HT1a with ^11^C‐CUMI tracer (*rho* = −0.574, *P* = 0.002) and 5HT2a with ^18^F‐ALTANSERIN tracer (*rho* = −0.529, *P* = 0.002) and ^11^C‐CIMBI tracer (*rho* = −0.533, *P* = 0.002) (Figure 6B).

### 2.11 Relationship between Morphological WM Networks and Gene Co‐Expression (AHBA Dataset)

Apart from the chemoarchitectonic covariance network, we constructed a gene co‐expression network to further explore potential neurobiological substrate of morphological WM networks. The gene co‐expression network was constructed by correlating regional gene expression profiles of 15633 genes among 38 WM ROIs (10 WM ROIs were excluded because no gene expression samples were assigned to them). The communication model was subsequently used to examine the ability of the group‐level mean morphological WM networks to predict the gene co‐expression network. These analyses were performed for the 6 participants in the AHBA dataset.

**Figure 7:**
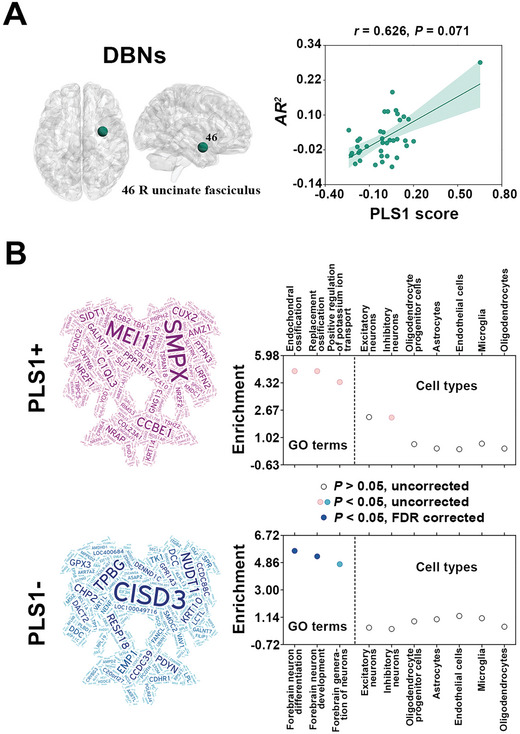
Relationship between morphological WM networks and gene co‐expression network. A) The DBNs significantly predicted the gene co‐expression profiles of the right uncinate fasciculus (*N* = 37 edges; multiple linear regression model, *P* < 0.05, FDR corrected; left panel). The spatial distribution pattern of regional *AR^2^* values tended to be explained by regional gene expression levels (*N *= 38 regions; PLS regression, *P* = 0.071; right panel). The number in the brain maps indicates the index of the identified region in the WM atlas from Johns Hopkins University. Dot size is proportional to regional *AR^2^* value. B) A total of 1442 genes were identified to show the strongest contributions to the PLS1 scores (PLS1+:837; PLS1‐: 605; left panel). Gene ontology enrichment analysis revealed that the PLS1‐ but not PLS1+ genes were enriched in specific biological processes (*P* < 0.05, FDR corrected; right panel). Neither the PLS1+ nor PLS‐ genes exhibited susceptibility to certain cell types (cell type‐specific aggregation analysis, *P* > 0.05, FDR corrected; right panel). *AR^2^*, adjusted R‐squared; DBNs, deformation‐based networks; FDR, false discovery rate; GO, gene ontology; PLS, partial least‐squares.

At the whole‐brain level, the VBNs (*AR^2^* = 0.045 *P* = 0.061) but not DBNs (*AR^2^* = 0.009, *P* = 0.439) significantly predicted the pattern of the gene co‐expression network. For node‐wise multilinear predictions, no significant predictions were observed for the VBNs (*AR^2^* = −0.080–0.288; *P* > 0.05, FDR corrected). For the DBNs (*AR^2^* = −0.079–0.281), a significant prediction was observed for the right uncinate fasciculus (*AR^2^* = 0.281, *P* < 0.001) (**Figure** **7**A, left).

We further linked the nodal *AR^2^* values for the DBNs (response variable) with regional expression levels of the 15633 genes (predictor variables) using the PLS regression. We found that the gene expression data explained marginally significant proportions of the variance in nodal *AR^2^* values for the DBNs (39.2%, *P* = 0.071) (Figure 7A, right). Analysis of the weights of the genes revealed a total of 837 and 605 genes that showed the strongest positive and nagative contributions to the PLS1 scores, respectively (i.e., PLS1+ and PLS1‐ genes). For the identified genes, we performed gene ontology (GO) enrichment analysis and cell type‐specific aggregation analysis. The PLS1‐ genes were found to be enriched in two biological processes of “forebrain neuron differentiation” and “forebrain neuron development”, whereas no biological processes were observed for the PLS1+ genes. Neither the PLS1+ nor PLS‐ genes exhibited susceptibility to certain cell types (*P* > 0.05) (Figure 7B).

### 2.12 Altered Morphological WM Networks in MS and NMOSD (MS and NMOSD Multicentric Dataset)

Finally, we evaluated the clinical correlates of morphological WM networks by examining their alterations in patients with MS and NMOSD. Notably, participants in this dataset were from seven different sites. Thus, we utilized a statistical harmonization method called Combat^[^ ^24^ ^]^ to remove site effects on the morphological WM networks. We examined the disease‐related alterations in morphological similarity [threshold‐free network‐based statistics approach (TFNBS)],^[^ ^25^ ^]^ association of altered morphological similarity with clinical variables (Spearman correlation), and classification of MS and NMOSD (binary linear support vector machine classifier).

#### 2.12.1 Altered Morphological Similarity

**Figure 8:**
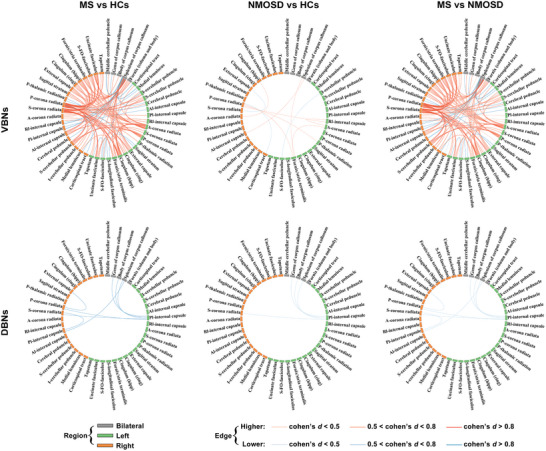
Edges showing significant between‐group differences in the MS and NMOSD multicentric dataset. A total of 106/10 edges were identified to exhibit significant group main effects on interregional morphological similarities in the VBNs/DBNs (*N* = 208 MS patients, 200 NMOSD patients, and 228 HCs; threshold‐free network‐based statistics approach, *P* < 0.05, family‐wise error corrected). See Table S4 (Supporting Information) for more details. VBNs, volume‐based networks; DBNs, deformation‐based networks; HCs, healthy controls; MS, multiple sclerosis; NMOSD, neuromyelitis optica spectrum disorders.

A total of 106 and 10 edges were identified to exhibit significant group main effects on interregional morphological similarities in the VBNs and DBNs, respectively (*P* < 0.05, family‐wise error corrected). Post hoc comparisons revealed that the edges exhibited MS‐specific alterations (VBNs: 99; DBNs: 8), common alterations to MS and NMOSD (VBNs: 5; DBNs: 2), or opposite alterations between MS and NMOSD (VBNs: 2) (**Figure** **8**; Table S3, Supporting Information).

#### 2.12.2 Association with Clinical Variables

**Figure 9:**
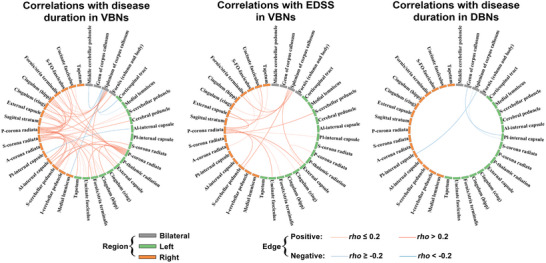
Edges showing significant correlations with clinical variables in the MS patients. For the VBNs, a total of 50/5 edges showed significant positive/negative correlations with disease duration (left panel) and a total of 21 edges showed significant positive correlations with EDSS scores (middle panel) of the MS patients (*N* = 208 MS patients; Spearman correlation, *P* < 0.05, false discovery rate corrected). For the DBNs, a total of 4 edges exhibited significant negative correlations with disease duration of the MS patients (*N* = 208 MS patients; Spearman correlation, *P* < 0.05, false discovery rate corrected; right panel). VBNs, volume‐based networks; DBNs, deformation‐based networks; EDSS, Expanded Disability Status Scale.

Among the edges in the VBNs showing altered morphological similarities in MS, 50/5 showed significant positive/negative correlations with disease duration and 21 showed significant positive correlations with Expanded Disability Status Scale scores of the patients (*P* < 0.05, FDR corrected). Out of the edges in the DBNs showing altered morphological similarities in MS, 4 exhibited significant negative correlations with disease duration of the patients (*P* < 0.05, FDR corrected) (**Figure** **9**; Table S3, Supporting Information). For the edges showing altered morphological similarities in NMOSD, no significant correlations were found with any clinical variables of the patients (*P* > 0.05, FDR corrected).

#### 2.12.3 Classification of MS and NMOSD

The VBNs distinguished the three groups from each other (MS vs controls: mean accuracy = 77.3%, *P* < 0.001; NMOSD vs controls: mean accuracy = 62.2%, *P* < 0.001; MS vs NMOSD: mean accuracy = 74.5%, *P* < 0.001). For the DBNs, the MS patients were distinguished from the controls (mean accuracy = 69.6%, *P* < 0.001) and NMOSD patients (mean accuracy = 60.5%, *P* < 0.001) while the classification between the NMOSD patients and controls failed (mean accuracy = 54.4%, *P* = 0.104).

### 2.13 Validation Results

#### 2.13.1 Consistency Across Different Datasets

To evaluate the cross‐dataset consistency of morphological WM networks, we calculated the Spearman rank correlations for the group‐level mean similarity matrix of morphological WM brain networks between each pair of the datasets included in this study except the JuSpace dataset. Moderate‐to‐high positive correlations were observed for both the VBNs (*rho* = 0.725–0.999) and DBNs (*rho* = 0.242–0.979) (Figure S2, Supporting Information). These findings indicate considerable consistency in the connectivity pattern of morphological WM brain networks across different datasets. Further comparison revealed that the correlations were significantly higher for the VBNs than DBNs (*P* < 0.001, permutation test), indicating higher cross‐dataset consistency for the VBNs than DBNs.

#### 2.13.2 Effects of Spatial Smoothing Kernel Size

In this study, individual WM volume and deformation maps were smoothed using a Gaussian kernel with 8‐mm full width at half maximum. Given that some WM ROIs were relatively small, we further evaluated the reproducibility of our results by employing smaller Gaussian kernel sizes for spatial smoothing (2, 4, and 6 mm). Specifically, we examined the similarity in the connectivity pattern (HCP dataset, unrelated participants) and the differences in the TRT reliability (HNU and SWU datasets) of morphological WM networks. We found that the group‐level mean similarity matrix of morphological WM networks derived from 8‐mm smoothed WM volume and deformation maps showed moderate‐to‐high positive Spearman rank correlations with those derived from WM volume and deformation maps smoothed with the smaller Gaussian kernel sizes (VBNs: *rho* = 0.434–0.879; DBNs: *rho* = 0.505–0.903; *P* < 0.05, FDR corrected). For the TRT reliability, most connections in both the VBNs and DBNs exhibited good‐to‐excellent TRT reliability irrespective of the choice of Gaussian kernel size (Table S4, Supporting Information). Nevertheless, morphological WM networks derived from 8‐mm smoothed WM volume and deformation maps showed significantly higher both short‐term and long‐term TRT reliability than those derived from WM volume and deformation maps smoothed with the smaller Gaussian kernel sizes (paired‐sample *T* test; *P* < 0.05, FDR corrected). These findings indicate that 8‐mm Gaussian kernel size is a reasonable choice for constructing morphological WM networks in this study.

#### 2.13.3 Effects of Age and Sex

To examine possible effects of age and sex on the phenotypic correlates of morphological WM networks, we reran the PLS regression and BBS modeling method after ruling out the effects of age and sex on morphological similarity of each edge and each behavioral and cognitive variable via a multiple linear regression model. We found that only the VBNs explained significant proportions of interindividual variance in the Cognition domain (15.1%, *P* = 0.007) and predicted individual scores of the Cognition domain (mean *r* = 0.155, *P* = 0.006) (Figure S3, Supporting Information). The association of morphological WM brain networks with the Motor domain was not found.

#### 2.13.4 Effects of Image Quality

Recent studies have shown that in‐scanner head motion is an important source of noise in brain morphometry studies.^[^ ^26^ ^]^ Thus, we further examined possible effects of head motion on our results. Given the lack of effective methods to quantify head motion from a single T1‐weighted MRI image, we used the Image Quality Rates (IQRs) generated by the CAT12 toolbox as a proxy for head motion since the amount of head motion during T1‐weighted MRI acquisition was previously demonstrated to be related to image quality of the acquired images.^[^ ^27^ ^]^ We first examined the relationship (Spearman rank correlation) between the IQRs and morphological WM networks (HCP dataset, unrelated participants). Significant correlations (TFNBS approach; *P* < 0.05, family‐wise error corrected) were observed between the IQRs and interregional morphological similarity for the VBNs (spatial smoothing: 69 edges; no spatial smoothing: 126 edges) and DBNs (spatial smoothing: 76 edges; no spatial smoothing: 111 edges) (Figure S4, Supporting Information). Then, we re‐analyzed the phenotypic correlates of morphological WM brain networks (HCP dataset, unrelated participants) after ruling out the effects of IQRs. We found that 1) the VBNs explained significant proportions of interindividual variance in the Cognition (15.1%, *P* = 0.005) and Motor (15.1%, *P* = 0.006) domains; 2) the VBNs predicted individual scores of the Cognition (mean *r* = 0.143, *P* = 0.014) and Motor (mean *r* = 0.224, *P* < 0.001) domains; 3) the DBNs predicted individual scores of the Motor domain (mean *r* = 0.163, *P* = 0.004) (Figure S5, Supporting Information). Finally, we re‐analyzed the clinical correlates of morphological WM networks (MS and NMOSD multicentric dataset) after ruling out the effects of IQRs for between‐group comparisons and correlation analyses. A total of 114 edges for the VBNs and 8 edges for the DBNs were identified to show significant group main effects on interregional morphological similarity (TFNBS approach; *P* < 0.05, family‐wise error corrected) (Figure S6, Supporting Information). Out of the 114/8 edges, 38/6 were correlated with disease duration (positive: 34/0; negative: 4/6) and 15/0 were correlated with Expanded Disability Status Scale scores (positive: 15/0; negative: 0/0) of the MS patients (*P* < 0.05, FDR corrected) (Figure S7, Supporting Information). These edges overlapped to a large extent with those in Figure 8 (VBNs: dice coefficient = 0.991; DBNs: dice coefficient = 1) and 9 (VBNs: dice coefficient = 0.835 for edges showing correlations with disease duration and 0.882 for edges showing correlations with Expanded Disability Status Scale scores; DBNs: dice coefficient = 0.667).

## 3 Discussion

In this study, we constructed volume‐ and deformation‐based morphological WM networks using structural MRI data, and systematically investigated their topological organization, TRT reliability, phenotypic association, heritability, functional and structural relevance, neurobiological substrate, and clinical value by combining multimodal and multiscale data. We found that morphological WM networks exhibited nontrivial topological organization, presented good‐to‐excellent TRT reliability, accounted for interindividual differences in cognition, were under genetic control, predicted WM hamodynamic coherence and metabolic synchronization, were associated with structural connectivity, predicted gene co‐expression and chemoarchitectonic covariance, and were able to help diagnose and differentiate MS and NMOSD. These findings deepen our understanding of the roles and origins of morphological WM networks and provide convincing evidence for the usage of morphological WM networks for individualized research of WM architecture in health and disease.

### 3.1 Specifically Organized Morphological WM Networks

Numerous studies have demonstrated the nontrivial topology of multimodal gray matter networks, such as small‐worldness, modularity, and rich‐club organization.^[^ ^3^, ^28^ ^]^ These organizational principles are recently found to exist in functional WM networks.^[^ ^5c^ ^]^ Here, we found that morphological WM networks were also specifically organized in an optimized manner to support efficient information spreading and processing. Moreover, the optimized topological organization was consistently observed regardless of the choice of morphological index and whether spatial smoothing was performed. These findings suggest that the optimized topological organization may be an intrinsic characteristic of morphological WM networks, presumably as a consequence of evolution by natural selection. Nevertheless, it should be noted that the DBNs and VBNs exhibited low correlations in their wiring patterns. Thus, these two types of networks are complementary to each other in the mapping of morphological WM networks. The low correlations may be due to fundamental differences in the nature between volume (reflecting a composite of thickness, area and folding) and deformation (reflecting local tissue expansion or contraction) and/or different neurobiological substrates between the VBNs and DBNs (see further discussion below).

### 3.2 TRT Reliable Morphological WM Networks

High TRT reliability is a prerequisite for a new method or measure before its use as potential clinical biomarkers. Here, we found that most edges in morphological WM networks exhibited good‐to‐excellent TRT reliability regardless of the scanning interval. These findings indicate that morphological WM networks can serve as a reliable approach to examine effects of interest on WM architecture. It should be noted that the TRT reliability of morphological WM networks was obviously higher than those reported for functional WM networks.^[^ ^4c^ ^]^ Therefore, relative to functional WM networks, morphological WM networks may be a preferable choice to study the wiring patterns of WM from the perspective of TRT reliability. Furthermore, we found that the TRT reliability of morphological WM networks depended on the choice of morphological index used for network construction (VBNs > DBNs) and can be improved by performing spatial smoothing. These findings are consistent with our previous findings from morphological gray matter networks^[^ ^8b,e^ ^]^ and provide guidance on determining analytical strategies for obtaining reliable morphological WM networks.

### 3.3 Behavioral and Cognitive Relevance of Morphological WM Networks

Numerous studies have demonstrated the tight association of multimodal gray matter networks with behavior and cognition.^[^ ^3^, ^9^, ^29^ ^]^ For functional WM networks, a previous study found that the small‐world organization was correlated with fluid intelligence in healthy participants.^[^ ^5c^ ^]^ Here, by combining multiple behavioral and cognitive domains with a multivariate approach, we showed that morphological WM networks were able to explain interindividual variance and predict individual scores for specific behavioral and cognitive domains. These findings are consistent with our previous study of morphological gray matter networks.^[^ ^9b^ ^]^ Specifically, both the VBNs and DBNs captured interindividual differences in the Motor domain. These findings sound plausible given the crucial roles of WM tracts in conveying motor information^[^ ^30^ ^]^ and previous findings that WM volume and deformation were related to motor performance.^[^ ^31^ ^]^ However, it should be noted that the association of morphological WM networks with the Motor domain became non‐significant after controlling for the effects of age and sex and thus should be explained with caution. In addition to the Motor domain, the VBNs were additionally associated with the Cognition domain. This is consistent with previous studies showing the relationship between WM volume and cognition.^[^ ^32^ ^]^ In view of this finding, the VBNs can be preferentially used to study neural correlates of cognition and brain diseases accompanied by cognitive dysfunction. Notably, all edges were used to examine the behavioral and cognitive association of morphological WM networks. Given previous findings from gray matter networks that different sets of highly interconnected nodes play domain‐specific roles in cognition,^[^ ^1^, ^33^ ^]^ it is interesting in the future to explore behavioral and cognitive relevance of morphological WM networks in the context of modular architecture.

### 3.4 Functional and Structural Correlates of Morphological WM Networks

In this study, we examined the functional and structural correlates of morphological WM networks by combining multimodal imaging data. We found that morphological WM networks were predictive of regional connectivity profiles derived from temporal fluctuations in hemodynamic activity and glucose metabolism. Moreover, the extent to which morphological WM networks explained variance of regional profiles of hamodynamic coherence followed the hierarchy of functional WM networks. This is consistent with previous findings from gray matter networks that the relationship between fiber tractography and hamodynamic coherence followed functional network hierarchy.^[^ ^34^ ^]^ It thus seems that the constraint on morphological/structural‐functional coupling from functional network hierarchy is a fundamental rule of the human brain regardless of gray matter and WM. It is worth noting that in contrast to the well characterized functional hierarchy of gray matter networks,^[^ ^35^ ^]^ significant gaps remain in our understanding of the functional hierarchy of WM networks with respect to its exact spatial pattern, developmental trajectory, and associations with other data modalities (e.g., gene expression and chemoarchitecture). Elucidating these issues can deepen our understanding of functional organization of WM networks, thereby helping explain the spatial pattern of functional correlates of morphological WM networks observed in this study. With respect to the structural correlates of morphological WM networks, no significant predictions were observed. Nevertheless, we found significant differences in WM morphological similarity between region pairs with and without structural connectivity. These findings suggest that there may be a complicated relationship between morphological and structural WM networks. Interestingly, an opposite pattern of the differences were observed between the VBNs and DBNs (i.e., higher volume‐based but lower deformation‐based morphological similarity for structurally connected region pairs). Actually, the functional correlates of morphological WM networks also exhibited a dissociation between the VBNs and DBNs. That is, the VBNs predicted regional profiles of metabolic synchronization, whereas the DBNs predicted regional profiles of hamodynamic coherence. These findings imply potentially distinct origins between the VBNs and DBNs. In the future, more sophisticated models may help more comprehensively elucidate the relationship of morphological WM networks with functional and structural WM networks.^[^ ^36^ ^]^

### 3.5 Neurobiological Substrate of Morphological WM Networks

In this study, we examined neurobiological substrate of morphological WM networks by linking them with genetic expression and chemoarchitecture. As for genetic basis, we first calculated edgewise heritability to show the extent to which morphological WM networks were heritable. We found that both the VBNs and DBNs were under genetic control with varying degrees across edges. Moreover, the heritability was significantly higher for the VBNs than DBNs. These findings indicate an important role of genes in determining morphological WM networks, particularly for the VBNs. Intriguingly, we found significant positive correlations for the heritability of edges in the VBNs with the edges’ contributions to accounting for interindividual differences in the Motor and Cognition domains. These correlations indicate that highly heritable edges in the VBNs are strongly correlated with individual motor and cognitive abilities. In addition to the heritability analysis, we examined the relationship between morphological and transcriptional WM networks. We found that the DBNs predicted gene co‐expression and the spatial distribution of regional predictive ability was related to a specific set of genes enriched in the forebrain neuron development and differentiation. The forebrain neuron development and differentiation support the progression of neurons in the forebrain from initial commitments to the final fully functional differentiated cells. A previous study found that initial axon extension started adjacent to a focus of microtubule polymerization within the cell body and the microtubules grew into the developing axon.^[^ ^37^ ^]^ Thus, the structural organization of the cell may establish the initial site and direction of axonal extension at the time of neuronal differentiation.^[^ ^38^ ^]^ Based on these findings, our results suggest that the forebrain neuronal progression may play an important role in the formation of the DBNs by modulating the growth of WM. It should be noted that despite under genetic control, the VBNs failed to predict gene co‐expression. This may be attributed to that the VBNs are relatively less involved in the genes used in this study.

In addition to the genetic basis, we examined the relationship between morphological and chemoarchitectonic WM networks. We found that the DBNs predicted chemoarchitectonic covariance between WM regions. The chemoarchitectonic basis was also found for morphological gray matter networks in our previous study.^[^ ^9b^ ^]^ Thus, it seems that chemoarchitecture may be a common source of morphological brain networks to both gray matter and WM. Furthermore, we found that regional predictive ability was correlated with the distribution of several serotonergic system‐related receptors (5HT1a and 5HT2a) and transporter (SERT). Serotonin has a diverse range of physiological roles, such as cell growth and differentiation,^[^ ^39^ ^]^ neuronal development,^[^ ^40^ ^]^ and neuronal signaling pathways. For WM, previous studies showed that the activation of 5HT1a and 5HT2a modulated the axonal excitability in rat spinal dorsal columns.^[^ ^41^ ^]^ For humans, the relationship between serotonin and WM was also increasingly reported. For instance, infants exposed to selective serotonin reuptake inhibitor were found to show increased WM structural connectivity.^[^ ^42^ ^]^ It should be noted that the physiological functions of neurotransmitter signaling in WM are debatable and further work is needed for deeper understanding the exact roles of chemoarchitecture in morphological WM networks.

### 3.6 Altered Morphological WM Networks in MS and NMOSD

To test the clinical value of morphological WM networks, we examined their alterations in MS and NMOSD, two diseases that are characterized by multifocal areas of WM lesions. We found that both diseases exhibited altered (increased and decreased) morphological similarity, particularly in the VBNs. These findings suggest that MS and NMOSD can be seen as diseases of morphological WM dysconnectivity. Nevertheless, much more alterations were observed in MS than NMOSD, indicative of more serious morphological WM dysconnectivity in MS. Moreover, many of the alterations in MS were correlated with disease duration and Expanded Disability Status Scale scores of the patients, highlighting the potential of morphological WM networks in monitoring disease progression of MS. Interestingly, both the alterations and correlations in MS were mainly involved in edges linking the splenium of corpus callosum and corona radiate, where the earliest WM atrophy occurred in the disease.^[^ ^43^ ^]^ These findings imply an important role of the splenium of corpus callosum and corona radiate in understanding the pathophysiology of MS. In the future, it is interesting to explore the relationship between WM atrophy and dysconnectivity as MS progresses. For NMOSD, fewer alterations were observed and most of the alterations were also identified in MS. These common alterations provide insights into shared neural mechanisms between MS and NMOSD from the perspective of morphological WM dysconnectivity. Notably, two edges in the VBNs exhibited opposite patterns in the alterations between MS and NMOSD. These results together with the findings that numerous edges in the VBNs were altered in MS but intact in NMOSD collectively suggest that relative to the DBNs, the VBNs may be a better choice in uncovering diagnosis‐specific biomarkers in MS and NMOSD. This speculation was supported by our classification analysis, which revealed higher accuracies for the VBNs than DBNs in distinguishing the three groups from each other. Notably, the classification accuracies were not that high, presumably due to the simple classification models used in this study. More sophisticated machine learning techniques and optimized model parameters may help improve the classification performance in the future.^[^ ^44^ ^]^

### 3.7 Limitations and Future Directions

There are several limitations and future directions that need to be considered. First, morphological WM networks were constructed separately based on WM volume and deformation maps derived from structural MRI. In addition to these two measures, WM morphology can be characterized by various microstructural descriptors from diffusion MRI, such as fractional anisotropy and mean diffusivity. Whether our method can be extended to these microstructural descriptors to map morphological WM brain networks is an open question. Second, accumulating evidence indicates that in‐scanner head motion causes systemic bias in morphological estimates from structural MRI,^[^ ^26^ ^]^ which further leads to alterations in morphological brain networks.^[^ ^27^ ^]^ Although our validation analyses indicated limited effects of head motion on our results, future studies are needed to develop methods to control for head motion effects on morphological WM networks. Third, effects of different analytical choices, including brain parcellation, connectivity estimation, and thresholding method have been well documented in the literature for multimodal gray matter networks.^[^ ^8^, ^45^ ^]^ Intuitively, these factors will also affect the topological characterization of morphological WM networks, which should be extensively studied in the future. Forth, this study only examined the association of morphological WM networks with WM networks derived from other data modalities. Beyond these cross‐modality comparisons of morphological WM networks, it is important to compare morphological brain networks between different brain tissues (i.e., WM vs gray matter).^[^ ^5c^ ^]^ For both types of comparisons, it is interesting for future studies to determine whether morphological WM networks have different sensitivities in uncovering alterations in brain disorders and different abilities in helping distinguish patients from controls. Fifth, the chemoarchitectonic WM network was constructed with publicly available data from various studies. This may result in an underestimation of the chemoarchitectonic correlates of morphological WM networks. Future work is needed to validate our results by collecting data from the same cohort of participants. Finally, we demonstrated the clinical value of morphological WM networks by applying them to MS and NMOSD. In addition to these two demyelinating diseases, future studies are required to examine whether morphological WM brain networks are capable of detecting alterations in psychiatric disorders, such as depression and schizophrenia. One thing that needs to be emphasized is that given the prevalence of multicentric clinical studies in recent studies, a significant step forward in the clinical application of morphological WM brain networks is to develop methods that are able to harmonize site effects for new samples from unseen scanners or sites.^[^ ^46^ ^]^

## 4 Conclusion

In summary, this study proposed an approach to construct morphological WM networks based on structural magnetic resonance imaging, and demonstrated that the morphological WM networks were TRT reliable, phenotypically related, genetically originated, functionally and structurally relevant, neurobiologically meaningful, and clinically valuable. The proposed approach provides an important way for studying WM in both health and disease.

## 5 Experimental Section

### Participants and Data Acquisition

This study included seven datasets. For each dataset, the participant recruitment processes and informed consent forms were ratified by the corresponding Institutional Review Board. This study was approved by the Institutional Review Board of the Institute for Brain Research and Rehabilitation at South China Normal University.

### HCP Dataset

The HCP dataset included 1113 participants with T1‐weighted structural images. In this study, a total of 444 unrelated healthy participants (age, 22–35 years; 205 males and 239 females) and 217 pairs of MZ and DZ twins (age, 22–35 years; 174 males and 260 females) were used. All MRI images were acquired using a customized 3T scanner at Washington University in St. Louis. The structural images were acquired using a magnetization‐prepared rapid gradient‐echo sequence with the following parameters: repetition time (TR) = 2,400 ms, echo time (TE) = 2.14 ms, flip angle (FA) = 8°, 256 slices, matrix = 320 × 320, field of view (FOV) = 224 × 224 mm^2^, slice thickness/gap = 0.7/0 mm, and voxel size = 0.7 × 0.7 × 0.7 mm^3^. The diffusion MRI data were acquired with a spin‐echo EPI sequence with the following parameters: TR = 5,520 ms, TE = 89.5 ms, FA = 78°, FOV = 210 × 180 mm^2^, voxel size = 1.25 × 1.25 × 1.25 mm^3^, and 270 non‐collinear directions with 3 non‐zero shells (b = 1000, 2000, and 3000 s mm^−2^). The resting‐state fMRI data were obtained using a multiband echo‐planar imaging sequence: TR =  720 ms, TE = 33 ms, FA = 52°, 72 slices, matrix = 104 × 90, FOV = 208 × 180 mm^2^, slice thickness/gap = 2/0 mm, and voxel size = 2 × 2 × 2 mm^3^. The resting‐state fMRI data were acquired in two sessions for each participant on consecutive days and each session consisted of two runs with left‐to‐right and right‐to‐left phase encoding protocols. The length of each resting‐state fMRI scan was 14.4 min (1200 volumes). In this study, only the resting‐state fMRI data with the left‐to‐right phase encoding in the first session were used. Notably, the diffusion MRI/resting‐state fMRI data were available only for 413/430 out of the 444 unrelated healthy participants.

In addition to the imaging data, the HCP dataset included a broad range of behavioral and cognitive measures that were evaluated mainly via the NIH Toolbox Assessment of Neurological and Behavioral function. For the initial 581 items of behavioral and cognitive measures, a multistep screening strategy was performed as in this study.^[^ ^9b^ ^]^ First, items related to health and family history, psychiatric and life function, and substance use were excluded. Items resulting from magnetoencephalogram and functional MRI tasks (e.g., accuracy and reaction time) were also deleted. For the remaining items, those that had the same values or were missing for more than 80% of the participants were further ruled out since they might fail to sufficiently capture interindividual differences. Furthermore, for items with unadjusted and adjusted values by age, original scores were used since using age‐adjusted versions has little effect on the relationships between brain networks and behavior.^[^ ^47^ ^]^ In addition, if total scores were provided for several items, the total scores were retained instead of individual items. After these procedures, a total of 60 items remained in this study, which were categorized into 6 behavioral and cognitive domains, including Alertness, Cognition, Emotion, Motor, Personality, and Sensory. Based on the 60 items, participants who did not complete any tests in at least one behavioral and cognitive domain were excluded from this study. To further exclude the effects of outliers, values that were 3.29 standard deviations above or below the mean for each item were replaced by the mean ± 3.29 standard deviations. Finally, the items were averaged within each domain after standardizing each item to make the scale comparable (Z‐transformation across participants).

### HNU Dataset

The HNU dataset included 30 participants (age, 20–30 years; 15 males and 15 females) that were scanned ten times over one month with one scan every three days. None of participants had a history of neurological or psychiatric disorders, substance abuse, or head injury with loss of consciousness. T1‐weighted structural images were obtained on a GE MR750 3.0 Tesla scanner (GE Medical Systems, Waukesha, WI) using a Fast Spoiled Gradient echo sequence: TR = 8.1 ms, TE = 3.1 ms, inversion time (TI) = 450 ms, FA = 8°, FOV = 256 × 256 mm^2^, matrix = 256 × 256, and voxel size = 1 × 1 × 1 mm^3^, and slices = 176.

### SWU Dataset

The SWU dataset contained 580 participants at baseline scan, some of whom completed the second (240) and third (228) scans. The average time intervals were 304.14, 515, and 817.87 days between the first and second scans, second and third scans, and first and third scans, respectively. All participants had no history of neurological or psychiatric disorders. T1‐weighted structural images were obtained on a 3T Siemens Trio MRI scanner using the magnetization‐prepared rapid gradient‐echo sequence: TR = 1,900 ms, TE = 2.52 ms, TI = 900 ms, FA = 9°, FOV = 256 × 256 mm^2^, matrix = 256 × 256, slice thickness = 1.0 mm, slices = 176, and voxel size = 1 × 1 × 1 mm^3^. In this study, a total of 120 participants (age, 17–22 years; 60 males and 60 females) who completed all three scans were included.

### MU Dataset

The MU dataset contained 27 participants (age, 18–23 years; 7 males and 20 females) who underwent a 95 min simultaneous MR‐PET scan in a Siemens (Erlangen) 3 Tesla Biograph molecular MR scanner (Syngo VB20 P). One participant (18 years old, female) was excluded from further analyses due to the poor performance of PET‐MRI co‐registration. All participants had no history of diagnosed Axis‐1 mental illness, diabetes, or cardiovascular illness. Over the course of the scan, [18‐F] fluorodeoxyglucose (average dose 233MBq) was infused at a rate of 36 mL hr^−1^ using a BodyGuard 323 MR‐compatible infusion pump (Caesarea Medical Electronics, Caesarea, Israel). The detailed protocols of resting‐state 18F‐fluorodeoxyglucose PET collection were listed elsewhere.^[^ ^16^ ^]^ T1‐weighted structural images were acquired on a 3T Siemens Biograph molecular MRI scanner using the magnetization‐prepared rapid gradient‐echo sequence: TR = 1,640 ms, TE = 2.34 ms, slices = 176, FOV = 256 × 256 mm^2^, FA = 8°, and voxel size = 1 × 1 × 1 mm^3^.

### JuSpace Dataset

The JuSpace dataset provided 27 neurotransmitter receptor and transporter maps from different studies on healthy participants, including 5HT1a, 5HT1b, 5HT2a, 5HT4, CB1, D1, D2, DAT, ^18^F‐DOPA, GABAa, mGluR5, MU, NAT, SERT, and VAChT. The D2 map derived from the raclopride tracer was excluded from further analysis because this tracer had unreliable binding in the cortex. Thus, a total of 26 neurotransmitter receptor and transporter maps were used in this study.

### AHBA Dataset

The AHBA dataset was a publicly available online resource of brain‐wide transcriptomic information and multimodal MRI obtained from six healthy adult human donors (age, 24–57 years; 5 males and 1 female) with no known history of neuropathological or neuropsychiatric disease.^[^ ^18^ ^]^ Specifically, transcriptional activity was recorded for 20737 genes from 3702 spatially distinct tissue samples that covered almost the entire brain. The tissue samples were collected from the left hemisphere for 4 donors and both hemispheres for 2 donors. Prior to dissection, T1‐weighted structural MRI images were acquired for each donor on a 3T Siemens Magnetom Trio scanners (Erlangen, Germany) using the MPRAGE sequence: TR = 1,900 ms, TI = 900 ms, and FA = 9°. Other imaging parameters differed among the donors. For more details, see http://human.brain‐map.org/.

### MS and NMOSD Multicentric Dataset

The MS and NMOSD multicentric dataset contained 208 MS patients, 200 NMOSD patients, and 228 healthy controls (age, 18–65 years; 104 males and 124 females for the controls, 73 males and 135 females for the MS patients, and 25 males and 175 females for the NMOSD patients). Details on the inclusion and exclusion criteria of patient selection and imaging parameters could be found in the previous study.^[^ ^19^ ^]^

### Preprocessing of Structural Images

All structural images underwent the same standard preprocessing pipeline using the CAT12 (http://www.neuro.uni‐jena.de/cat/) based on the SPM12 package (https://www.fil.ion.ucl.ac.uk/spm/software/spm12). Briefly, each structural image was first segmented into gray matter, WM, and cerebrospinal fluid using an adaptive Maximum A Posterior technique. The resulting WM maps were then normalized to the standard Montreal Neurological Institute (MNI) space using a geodesic shooting approach.^[^ ^48^ ^]^ WM volume was calculated by modulating the WM maps using Jacobian determinants derived from the spatial normalization. Finally, a WM volume map and deformation map (i.e., Jacobian determinants) were obtained for each structural image. Of note, the Segment Longitudinal Data module in the CAT12 was used for tissue segmentation of structural images in the SWU and HNU datasets. In addition, a WM lesion mask was manually delineated for each patient in the MS and NMOSD multicentric dataset using the 3D‐slicer software (https://www.slicer.org) if WM hyperintensity was evident on the T2‐weighted FLAIR image. Based on the resulting masks, a superior longitudinal fasciculus algorithm (http://atc.udg.edu/salem/slfToolbox/software.html) was used to refill WM lesions on individual T1‐weighted images before image preprocessing.

### Spatial Smoothing

Spatial smoothing was frequently used to increase the signal‐to‐noise ratio, and improve interindividual anatomical correspondence for voxel‐based morphology analysis. However, it might introduce spurious local connectivity between spatially adjacent regions. In the previous study, it was found that spatial smoothing significantly affected the wiring patterns and topological organization of morphological brain networks between gray matter regions, and the implementation of spatial smoothing increased the TRT reliability.^[^ ^8b^ ^]^ To evaluate the effects of spatial smoothing on morphological WM networks, individual WM volume and deformation maps with (Gaussian kernel with 8 mm full width at half maximum) and without spatial smoothing were separately used to construct morphological WM networks.

### Construction of Morphological WM Networks

Morphological WM networks were constructed using the previous approaches for depicting morphological brain networks between gray matter regions.^[^ ^8b,e^ ^]^ First, a WM atlas from Johns Hopkins University^[^ ^12^ ^]^ was used to divide brain WM into 48 ROIs. Then, values of WM volume and deformation were separately extracted from all voxels within each ROI to derive regional probability density functions (MATLAB function, ksdensity). The resulting probability density functions were further transformed into probability distributions (PDs). Subsequently, interregional morphological similarity was estimated as:

(1) $$JSDs\left(P,Q\right)=1-\sqrt{JSD(P||Q)}$$

(2) $$JSD\left(P||Q\right)=\frac{1}{2}\;\sum_{i=1}^{n}P\left(i\right)\log\frac{P\left(i\right)}{M\left(i\right)}+\frac{1}{2}\sum_{i=1}^{n}Q\left(i\right)\log\frac{Q\left(i\right)}{M\left(i\right)}$$

(3) $$M=\frac{1}{2}\left(P+Q\right)$$

where *P* and *Q* denote regional PDs, and *n* denotes the number of sampling points during the process of probability density estimation. The value of JSDs indicates the extent to which two PDs are similar (0 indicating completely different and 1 exactly the same). Finally, a total of four morphological WM networks were obtained for each structural image [2 morphological indices (volume vs deformation) × 2 choices of spatial smoothing (yes vs no)].

In this study, the number of sampling points during the process of probability density estimation was set to 2^8^ to align with the previous study of morphological brain networks of gray matter.^[^ ^8b^ ^]^ To examine whether this parameter setting was applicable to regional probability density estimation for WM volume and deformation, the effects of the number of sampling points (HCP dataset, unrelated participants) were evaluated using the same method as that used in the previous study.^[^ ^8b^ ^]^ Briefly, the Frechet distance was first calculated for each WM ROI to quantify the similarity of its probability density functions that were estimated using different numbers of sampling points (2^4^ vs 2^5^, 2^5 ^vs 2^6^, 2^6 ^vs 2^7^, 2^7 ^vs 2^8^, 2^8 ^vs 2^9^, and 2^9^ vs 2^10^). This resulted in a 444 (participants) × 48 (ROIs) × 6 (pairs of adjacent sampling number) Frechet distance matrix separately for smoothed WM volume, unsmoothed WM volume, smoothed WM deformation, and unsmoothed WM deformation. Each Frechet distance matrix was subsequently averaged across participants to derive a group‐level Frechet distance matrix (48 × 6). Finally, nonparametric permutation tests (10000 times) were used to compare any two adjacent columns in each group‐level Frechet distance matrix. It was found that the similarity between regional probability density functions significantly and continuously increased with the increasing number of sampling points (*P* < 0.05, FDR corrected) regardless of the choice of morphological index and whether spatial smoothing was performed (Table S5, Supporting Information). These findings suggest that more sampling points were associated with more stable estimation of regional probability density functions. However, to avoid overfitting and to provide a trade‐off between stable estimation and computational cost, 2^8^ was chosen in this study.

### Effects of Different Analytical Strategies on Morphological WM Networks (HCP Dataset, Unrelated Participants)

To explore the effects of morphological index and spatial smoothing on morphological WM networks, the Spearman correlation between the group‐level mean VBN and DBN, and the Spearman correlation of the group‐level mean morphological WM network between data with and without spatial smoothing was first calculated. Significance levels of the correlations were estimated by simulating morphological similarity matrices via Moran spectral randomization to account for spatial autocorrelation (10000 times).^[^ ^49^ ^]^ Furthermore, two‐way repeated ANOVA was performed on the group‐level mean morphological similarity of all edges in morphological WM networks. For significant effects, post hoc comparisons were further performed with paired‐sample *T* tests.

### Topological Analysis of Morphological WM Networks (HCP Dataset, Unrelated Participants)—Threshold Selection

Before topologically characterizing morphological WM networks, a sparsity‐based thresholding procedure was first used to convert them to binary networks. Sparsity is defined as the ratio of the number of actual edges to the maximum possible number of edges in a network. By applying subject‐specific thresholds to morphological WM networks, the sparsity‐based thresholding procedure ensures the same number of edges across participants and visits under different analytical strategies. Given the lack of a definitive standard for choosing a single sparsity, a consecutive sparsity range of [0.09 0.3] with an interval of 0.02 was used in this study. This sparsity range was determined to guarantee that the resulting binary networks were sparse and estimable for the small‐world attributes.^[^ ^45^, ^50^ ^]^ To ensure that there were no isolated nodes or multiple connected components in the resulting binary networks, a minimum spanning tree algorithm^[^ ^51^ ^]^ was further integrated into the sparsity‐based thresholding procedure. For the resulting binary networks, several graph‐based network measures were calculated with the GRETNA toolbox.^[^ ^52^ ^]^ Detailed formulas and interpretations of the network measures could be found elsewhere.^[^ ^53^ ^]^

### Small‐Worldness and Rich‐Club Organization

The small‐world attributes (clustering coefficient and characteristic path length) and rich‐club coefficient for each morphological WM network at each sparsity was calculated. To test whether morphological WM networks were non‐randomly organized, these global measures were further normalized by the corresponding mean of 100 matched random networks, which were generated with a topological rewiring algorithm to preserve the same degree distribution as the actual networks.^[^ ^54^ ^]^ Typically, a small‐world network should exhibit a normalized clustering coefficient > 1 and a normalized characteristic path length ≈1^[^ ^50b^ ^]^ and a network with rich‐club organization should exhibit a normalized rich‐club coefficient > 1.^[^ ^53c^ ^]^

### Degree Distribution and Hubs

To characterize the roles of individual nodes in morphological WM networks, nodal degree was calculated for each morphological WM network at each sparsity. After averaging nodal degree across participants and over different sparsity levels, different models (power law, exponential, and exponentially truncated power law) were used to fit the degree distribution of the morphological WM networks. Regions with a Z‐transformed mean nodal degree > 1 were identified as hubs.

### TRT Reliability of Morphological WM Networks (HNU and SWU Datasets)

The *ICC* was used to quantify both short‐term (HNU dataset) and long‐term (SWU dataset) TRT reliability of morphological WM networks.^[^ ^55^ ^]^ Formally, for each edge in morphological WM networks, the *ICC* was calculated as:

(4) $$ICC=\frac{MS_{b}-\;MS_{w}}{MS_{b}+\left(k-1\right)MS_{w}}$$

where *MS_b_* is the mean square of between‐subject variance, *MS_w_* is the mean square of within‐subject variance, and *k* denotes the number of repeated observations per participant (10 for the HNU dataset and 3 for the SWU dataset). *ICC* is close to 1 for reliable measures and 0 otherwise. In accordance with the previous studies,^[^ ^8b,e^ ^]^ the TRT reliability was categorized as poor (*ICC* < 0.25), low (0.25 < *ICC* < 0.4), fair (0.4 < *ICC* < 0.6), good (0.6 < *ICC* < 0.75), or excellent (0.75 < *ICC* < 1).

### Effects of Different Analytical Strategies on TRT Reliability of Morphological WM Networks (HNU and SWU Datasets)

To examine the effects of morphological index and spatial smoothing on the TRT reliability of morphological WM networks, two‐way repeated ANOVA was performed separately on the short‐term and long‐term TRT reliability of all edges in morphological WM networks. For significant effects, post hoc comparisons were further performed with paired‐sample *T* tests.

### Behavioral and Cognitive Correlates of Morphological WM Networks (HCP Dataset, Unrelated Participants)

The previous study found that morphological brain networks of gray matter were able to explain interindividual variance and predict individual scores for specific behavioral and cognitive domains.^[^ ^9b^ ^]^ Here, it was further examined whether this ability was shared by morphological WM networks. Specifically, the PLS regression was used to examine the relationship between morphological WM networks and behavioral and cognitive data at each domain. In the PLS regression model, the response variable was behavioral and cognitive data for a certain domain and the predictor variables were morphological similarity of all edges in morphological WM networks. The PLS1 was the linear combination of interregional morphological similarity that exhibited the strongest correlation with the behavioral and cognitive data. Significance levels of the correlations were estimated by randomly shuffling the behavioral and cognitive data among participants (10000 times). The FDR procedure was used to correct for multiple comparisons across all behavioral and cognitive domains at the level of *q* < 0.05 for the VBNs and DBNs, respectively. For each significant correlation, the contribution of a given edge was defined as its weight to form the PLS1. In addition to the PLS regression, a BBS modeling method^[^ ^20^ ^]^ was used to explore whether morphological WM networks were predictive of individual scores of each behavioral and cognitive domain. First, principal component analysis was used for dimensionality reduction by retaining components that explained 80% variance in interregional morphological similarity. Then, a linear regression model was fitted between the expression scores of the retained components and behavioral and cognitive data for each domain, which was further used to predict behavioral and cognitive outcomes for unseen participants. Finally, the Pearson correlation between actual scores and predicted values was calculated for each behavioral and cognitive domain. To assess the performance of the BBS model, a ten‐fold cross‐validation procedure was used. Since a single cross‐validation might be sensitive to a particular split of the data into folds,^[^ ^56^ ^]^ the ten‐fold cross‐validation procedure was repeated 100 times and the resulting mean Pearson correlation coefficient was reported for each behavioral and cognitive domain. To test whether the Pearson correlation coefficients were significantly higher than random operations, a nonparametric permutation testing procedure was performed by reshuffling the behavioral and cognitive data and repeating the ten‐fold cross‐validation procedure (10000 times). The FDR procedure was used to correct for multiple comparisons across all behavioral and cognitive domains at the level of *q* < 0.05 for the VBNs and DBNs, respectively. For each significant correlation, the contribution of a given edge was calculated as the mean value of the product of the coefficient in principal component analysis with the beta value in the linear regression model across all folds and repetitions.

### Heritability of Morphological WM Networks (HCP Dataset, Twin Participants)

A genetic ACE model was used to investigate the extent to which morphological WM networks were genetically controlled. In a genetic ACE model, the variance of a phenotypic variable is assumed to be the sum of additive genetic contribution (A) and common (C) and unique environment (E) contribution.^[^ ^57^ ^]^ Formally, the narrow‐sense heritability was defined as the proportion of phenotypic variance that was attributed to genetic factors:

(5) $$h^{2}=\frac{A}{A+C+E}$$

The heritability was estimated for each edge in morphological WM networks with the APACE package.^[^ ^58^ ^]^ Significance level of each edge's heritability was estimated by randomly shuffling the labels (MZ or DZ) of each pair of twins (10000 times) to generate an empirical distribution. The FDR procedure was used to correct for multiple comparisons across all edges at the level of *q* < 0.05 for the VBNs and DBNs, respectively.

The relationship between the heritability of edges in morphological WM networks and the contributions of the edges to the phenotypic correlates of morphological WM networks was further examined via Spearman correlation. Significance levels of the correlations were estimated by simulating the heritability matrix via Moran spectral randomization to account for spatial autocorrelation (10000 times).^[^ ^49^ ^]^ The FDR procedure was used to correct for multiple correlations at the level of *q* < 0.05 for the VBNs and DBNs, respectively.

### Hamodynamic Correlates of Morphological WM Networks (HCP Dataset, Unrelated Participants)—Preprocessing of Resting‐State fMRI Images

For individual resting‐state fMRI images, the HCP fMRIVolume pipeline was used to generate minimally preprocessed 4D time series, including gradient distortion correction, motion correction, field map‐based echo‐planar imaging distortion correction, registration of functional data to structural scan, non‐linear registration into the standard MNI space, and grand‐mean intensity normalization.^[^ ^59^ ^]^ After these steps, individual resting‐state fMRI images further underwent band‐pass filtering (0.01 to 0.08 Hz) and removal of nuisance covariates [24‐parameter head motion profiles,^[^ ^60^ ^]^ cerebrospinal fluid signals, and global signals] in a single regression model to avoid reintroducing artifacts.^[^ ^61^ ^]^ The cerebrospinal fluid signals were calculated according to a prior cerebrospinal fluid probability map released in the SPM12 package (threshold = 0.9).

### Construction of Hamodynamic WM Networks

The hamodynamic WM networks were constructed using the same WM parcellation atlas as for morphological WM networks. First, regional mean time series was extracted for each ROI by averaging signals of all voxels within the ROI for each volume. The resulting time series were then correlated with each other to generate a hamodynamic coherence matrix for each participant. Finally, the hamodynamic coherence matrices were averaged across participants to derive a group‐level mean hamodynamic WM network.

### Relationship between Morphological and Hamodynamic WM Networks

The communication model was used, a multiple linear regression model based on simple dynamical processes,^[^ ^21^ ^]^ to predict the group‐level mean hamodynamic coherence network with the group‐level mean morphological WM network and group‐level mean networks of communicational organization (i.e., shortest path length and communicability). Specifically, the communicational organization of a node combined both centralized (shortest path length) and decentralized (communicability) policies that required global and local knowledge of a network's topology, respectively.^[^ ^21^ ^]^ Shortest path length was calculated as the minimum number of edges required to go from one node to another and communicability was defined as the weighted sum of all paths and walks between two nodes.^[^ ^62^ ^]^ Shortest path length and communicability were both estimated from individual binary morphological WM networks and averaged across participants. The *AR^2^* value was used to assess the performance of the multiple linear regression model, which was constructed at the whole‐brain level and for each node. Significance levels of the *AR^2^* values were estimated by predicting the hamodynamic coherence simulated via Moran spectral randomization (10000 times).^[^ ^49^ ^]^ The FDR procedure was used to correct for multiple comparisons across all nodes at the level of *q* < 0.05 for the VBNs and DBNs, respectively.

### Association of the Relationship between Morphological and Hamodynamic WM Networks with Functional Hierarchy

To explore whether the relationship between morphological and hamodynamic WM networks follows functional hierarchy, the gradients of the hamodynamic WM network were calculated using the BrainSpace toolbox^[^ ^22^ ^]^ in a similar way as for functional networks of gray matter.^[^ ^63^ ^]^ Briefly, an affinity matrix was first derived by calculating the cosine similarity between each pair of regional group‐level mean hamodynamic coherence profiles that contained the top 10% of correlations for each node. Then, diffusion map embedding, a nonlinear dimensionality reduction technique, was used to identify principle gradients that accounted for primary variations in the distribution of hamodynamic coherence across different regions. Finally, the Spearman rank correlation between the nodal *AR^2^* values derived above and the first gradient of the hamodynamic WM network (Z‐transformed) was calculated. Significance level of the correlation was estimated by simulating the nodal *AR^2^* values via Moran spectral randomization to account for spatial autocorrelation (10000 times).^[^ ^49^ ^]^

### Metabolic Correlates of Morphological WM Networks (MU dataset)—Preprocessing of Resting‐State PET Images

The resting‐state PET images were preprocessed using the SPM12 package (https://www.fil.ion.ucl.ac.uk/spm/software/spm12/). First, the 225 volumes commencing from the 30 min time point were retained, which matched the start of the resting‐state fMRI data acquisition in the dataset.^[^ ^16^ ^]^ Then, individual resting‐state PET images were corrected for head motion and normalized into the standard MNI space via transformation fields derived from tissue segmentation of structural images. One participant was excluded from further analyses due to the poor performance of PET‐MRI co‐registration. Finally, individual resting‐state PET images were processed using a spatiotemporal gradient filter, the convolution of a 3D Gaussian filter in the spatial domain (standard deviation, one voxel), and a 1D Gaussian filter in the time domain (standard deviation, two frames), to estimate short‐term changes in glucose uptake from the cumulative glucose uptake that was measured.^[^ ^16^ ^]^

### Construction of Metabolic WM Networks

The metabolic WM networks were constructed using the same WM parcellation atlas as for morphological WM networks. First, regional mean time series was extracted for each ROI by averaging signals of all voxels within the ROI for each volume. The resulting time series were then correlated with each other to generate a metabolic synchronization matrix for each participant. Finally, the metabolic synchronization matrices were averaged across participants to derive a group‐level mean metabolic WM network.

### Relationship between Morphological and Metabolic WM Networks

The relationship between morphological and metabolic WM networks was examined using the multiple linear regression model in the same manner as for the relationship between morphological and hamodynamic WM networks.

### Association of the Relationship between Morphological and Metabolic WM Networks with Regional Metabolism

To test whether the relationship between morphological and metabolic WM networks was related to regional metabolism, the Spearman rank correlation between nodal *AR^2^* values and regional static metabolic values was calculated. For a given region, the static metabolic value was calculated as the mean metabolic activity across all time points and participants. Significance level of the correlation was estimated by simulating the nodal *AR^2^* values via Moran spectral randomization to account for spatial autocorrelation (10000 times).^[^ ^49^ ^]^

### Structural Correlates of Morphological WM Networks (HCP Dataset, Unrelated Participants)—Preprocessing of Diffusion MRI Images

For individual diffusion MRI images, the HCP diffusion preprocessing pipeline was used to generate minimally preprocessed diffusion MRI data, including b0 image intensity normalization across runs, EPI distortion correction, eddy current and motion correction, gradient nonlinearity correction, registration to native structural space, and B1 field inhomogeneity correction.^[^ ^59^ ^]^

### Construction of Structural WM Networks

Based on the preprocessed diffusion MRI data, individual structural WM brain networks were obtained using reconstructed whole‐brain WM tracts as in the previous study.^[^ ^64^ ^]^ Briefly, the mrtrix_multishell_msmt_ACT‐hsvs  method in MRtrix3 was used to reconstruct whole‐brain WM tracts, which implemented a multi‐shell, multi‐tissue constrained spherical deconvolution to estimate the fiber orientation distribution for each voxel.^[^ ^65^ ^]^ During the process of fiber reconstruction, an anatomically constrained tractography framework was used to improve the biological accuracy.^[^ ^66^ ^]^ Based on the reconstructed whole‐brain WM tracts, individual structural connectivity matrices were constructed using the command tck2connectome with the WM atlas from Johns Hopkins University as an a priori parcellation scheme. The weight of structural connectivity between two regions was the number of streamlines connecting the two regions, which was further normalized by the average volume of the two regions.^[^ ^67^ ^]^

### Relationship between Morphological and Structural WM Networks

The relationship between morphological and structural WM networks were examined using the multiple linear regression model in the same manner as for the relationship between morphological and hamodynamic WM networks. Given that structural WM brain networks contained many zeros, which may affect the performance of the multiple linear regression model, morphological similarity between region pairs with and without structural connectivity (paired‐sample *T* test) was also directly compared.

### Chemoarchitectonic Correlates of Morphological WM Networks (JuSpace Dataset and HCP Dataset, Unrelated Participants)—Construction of Chemoarchitectonic WM Network

The chemoarchitectonic WM network was constructed using the same WM parcellation atlas as for morphological WM networks. First, the mean intensity was extracted for each ROI from each neurotransmitter receptor and transporter map in the JuSpace dataset. The resulting regional profiles were then Z‐transformed to make comparability across different neurotransmitter receptors and transporters. Finally, the Z‐transformed regional neurotransmitter receptor and transporter profiles were correlated with each other to generate a chemoarchitectonic covariance matrix.

### Relationship between Morphological and Chemoarchitectonic WM Networks

The relationship between morphological and chemoarchitectonic WM networks was examined using the multiple linear regression model in the same manner as for the relationship between morphological and hamodynamic WM networks.

Association of the relationship between morphological and chemoarchitectonic WM networks with regional neurotransmitter intensity. To investigate whether the relationship between morphological and chemoarchitectonic WM networks was related to regional intensity of certain neurotransmitter receptor and transporter, the Spearman rank correlation between nodal AR^2^ values and regional mean intensity of each neurotransmitter receptor and transporter was calculated. Significance levels of the correlations were estimated by simulating the nodal AR^2^ values via Moran spectral randomization to account for spatial autocorrelation (10000 times).^[^ ^49^ ^]^ The FDR procedure was used to correct for multiple comparisons across all neurotransmitter receptors and transporters at the level of *q* < 0.05 for the VBNs and DBNs, respectively.

### Genetic Correlates of Morphological WM Networks (AHBA Dataset)—Preprocessing of Gene Data

Standardized workflows^[^ ^68^ ^]^ were used to preprocess gene data in the AHBA dataset with the abagen toolbox (version 0.1.3; https://github.com/rmarkello/abagen).^[^ ^69^ ^]^ First, the probe‐to‐gene annotations were updated using up‐to‐date information. Then, intensity‐based filtering was applied to exclude probes that did not exceed background noise in more than 50% of the samples. Afterward, a representative probe was selected for each gene that had the most consistent pattern of regional variations across the six donor brains as quantified by a measure called Differential Stability.^[^ ^70^ ^]^ To assign gene expression samples to the WM ROIs, samples further than 2 mm away from any voxel in the parcellation were excluded and assigned each of the remaining samples to its nearest ROI according to the minimum distance between the sample and any voxel in a ROI. Ten WM ROIs were excluded because no samples were assigned to them. Finally, gene expression levels of the remaining samples were normalized for each donor by applying a scaled robust sigmoid normalization to every sample across genes and to every gene across samples in order to assess the relative expression of each gene across regions while controlling for donor‐specific differences in gene expression. After these procedures, the gene expression profiles of 15633 genes were obtained for 38 WM ROIs.

### Construction of Transcriptional WM Network

The transcriptional WM network was constructed using the same WM parcellation atlas as for morphological WM networks. First, the mean expression level of each gene was extracted for each ROI by averaging across samples of all donors assigned to the same ROI. The resulting regional gene expression profiles were then correlated with each other to generate a transcriptional gene co‐expression network.

### Relationship between Morphological and Transcriptional WM Networks

The relationship between morphological and transcriptional WM networks were examined using the multiple linear regression model in the same manner as for the relationship between morphological and hamodynamic WM networks.

### Association of the Relationship between Morphological and Transcriptional WM Networks with Regional Gene Expression

To investigate whether the relationship between morphological and transcriptional WM networks was related to regional expression of certain genes, the PLS regression was performed to correlate nodal *AR^2^* values with regional expression levels of all genes. The PLS1 was the linear combination of regional expression levels of all genes that exhibited the strongest correlation with the nodal *AR^2^* values. Significance level of the correlation was estimated by re‐running the PLS regression for nodal *AR^2^* values simulated via Moran spectral randomization to account for spatial autocorrelation (10000 times).^[^ ^49^ ^]^ If a significant correlation was observed, the weights of all genes to form the PLS1 were Z‐transformed. Genes with an absolute Z‐score > 1.64 were considered to strongly contribute to relationship between the morphological and transcriptional WM networks.

### Gene Ontology Enrichment Analysis

For the identified genes that strongly contributed to the relationship between morphological and transcriptional WM networks, GO enrichment analysis was performed to search for their related GO terms. First, the biological process‐related GO term hierarchy files and annotation files were downloaded for Homo sapiens (version April 17, 2019) from https://figshare.com/s/71fe1d9b2386ec05f421.^[^ ^71^ ^]^ Then, the gene‐to‐category annotations were ran, processed the hierarchy relationships between GO terms, and restricted the analysis to the GO terms with 10–1000 gene annotations.^[^ ^72^ ^]^ To reduce the false‐positive rate in the GO enrichment analysis, a spatial ensemble null model was used.^[^ ^71^ ^]^ Specifically, for each of the resulting GO terms, an enrichment coefficient was calculated. To estimate the significance levels of the enrichment coefficients, the PLS regression was re‐ran for nodal *AR^2^* values simulated via Moran spectral randomization^[^ ^49^ ^]^ and re‐calculated the enrichment coefficient for each GO term. These procedures were repeated 10000 times to generate a null distribution for each GO term. Based on the null distribution, a *P*‐value was calculated as the proportion of repetitions, for which the resulting enrichment coefficient exceeded or equaled the real observation. Significant GO terms were determined after correcting for multiple comparisons with the FDR procedure at the level of *q* < s0.05. Notably, the GO enrichment analysis was performed separately for genes that positively and negatively contribute to the relationship between morphological and transcriptional WM networks.

### Cell Type‐Specific Aggregation Analysis

For the identified genes that strongly contributed to the relationship between morphological and transcriptional WM networks, the effects of regional variations in cellular architecture on their contributions were explored. First, an enrichment coefficient was calculated for each of the seven canonical cell classes: excitatory neurons, inhibitory neurons, oligodendrocyte progenitor cells, astrocytes, endothelial cells, microglia, and oligodendrocytes.^[^ ^73^ ^]^ The significance levels of the enrichment coefficients were then estimated in a similar manner as for the GO enrichment analysis. Finally, the FDR procedure was used to correct for multiple comparisons across all cell classes at the level of *q* < 0.05. Again, the cell type‐specific aggregation analysis was performed separately for the PLS1+ and PLS1‐ genes.

### Clinical Correlates of Morphological WM Networks (MS and NMOSD Multicentric Dataset)

Clinical correlates of morphological WM networks were explored by examining disease‐related alterations in interregional morphological similarity, association of altered morphological similarity with clinical variables, and classification and differentiation of MS and NMOSD. Before these analyses, morphological WM networks were harmonized for site effects using the ComBat (https://github.com/Jfortin1/ComBatHarmonization/tree/master/Matlab).

### Disease‐Related Alterations

The TFNBS approach^[^ ^25^ ^]^ was used to examine the differences in interregional morphological similarity among the three groups. Specifically, one‐way ANCOVA was first performed for each edge in morphological WM networks with the group as a between‐subject factor and sex and age as covariates. This resulted in a 48 × 48 *F*‐statistic matrix, which was then enhanced with the TFNBS approach by combining the threshold‐free cluster enhancement and network‐based statistic.^[^ ^74^ ^]^ The extension and height enhancement parameters were set to 0.5 and 2.25, respectively, based on the recommendations from Baggio and colleagues.^[^ ^25^ ^]^ Significance levels of the enhanced *F* statistics were estimated through a nonparametric permutation testing procedure (10000 times) and were corrected for multiple comparisons by comparing each edge's statistic to the distribution of the maximum statistic of all edges under the null hypothesis. For edges showing significant group effects, post hoc tests were further performed using the TFNBS approach based on independent‐sample *T* test.

### Association with Clinical Variables

Spearman rank correlation was used to examine the relationship between the altered morphological similarity and clinical variables (disease duration and Expanded Disability Status Scale score) in each patient group. Effects of age and sex were removed from the altered morphological similarity via a multiple linear regression model. The FDR procedure was used to correct for multiple comparisons in each patient group at the level of *q* < 0.05.

### Classification and Differentiation

To examine the potential of morphological WM networks in diagnosing and differentiating MS and NMOSD, a binary linear support vector machine classifier was trained for each pair of groups with all edges in morphological WM networks as original features. Before constructing the classifiers, principal component analysis was used for dimensionality reduction by retaining components that explained 80% variance in the original features. To assess the performance of the classifiers, a ten‐fold cross‐validation procedure was used. Since a single cross‐validation might be sensitive to a particular split of the data into folds,^[^ ^56^ ^]^ the ten‐fold cross‐validation procedure was repeated 100 times and the resulting mean accuracy was reported for the classification. To test whether the classification accuracies were significantly higher than random operations, a nonparametric permutation testing procedure was performed by reshuffling the group labels of participants and repeating the ten‐fold cross‐validation procedure (10000 times).

### Validation Analysis—Consistency Across Different Datasets

In this study, morphological WM networks were constructed for six independent datasets to answer different questions. To examine the consistency in the morphological WM networks across these datasets, the Spearman rank correlations were calculated for the group‐level mean similarity matrix of morphological WM networks between each pair of the datasets. Specifically, for the HCP dataset, the mean similarity matrix was calculated for the unrelated healthy participants and twin participants, respectively; for the HNU dataset, the mean similarity matrix was calculated for each of the ten scans; for the SWU dataset, the mean similarity matrix was calculated for each of the three scans; and for the MS and NMOSD multicentric dataset, the mean similarity matrix was calculated for the healthy controls. Significance levels of the correlations are estimated through Moran spectral randomization to account for spatial autocorrelation (10000 times).

### Effects of Spatial Smoothing Kernel Size

In this study, an 8 mm Gaussian kernel size was used for spatial smoothing of WM volume and deformation maps. This parameter setting was consistent with previous voxel‐based morphometry studies on WM.^[^ ^75^ ^]^ In particular, 8‐mm Gaussian kernel size was chosen a priori for voxel‐based morphometry studies on WM in the recently developed tool that was specifically designed for the ENIGMA consortium.^[^ ^76^ ^]^ However, this parameter setting may be too large given the relatively small size of some ROIs in the WM atlas from Johns Hopkins University. Thus, morphological WM networks were re‐constructed using WM volume and deformation maps that were smoothed with smaller smoothing kernel sizes (2, 4, and 6 mm). The resulting networks were compared with those derived from WM volume and deformation maps smoothed with 8‐mm Gaussian kernel with respect to the connectivity pattern (HCP dataset, unrelated participants; Spearman rank correlation) and TRT reliability (HNU and SWU datasets; paired‐sample *T* test).

### Effects of Age and Sex

Age and sex are important influencing factors for behavior and cognition.^[^ ^77^ ^]^ Thus, the phenotypic correlates of morphological WM networks were re‐analyzed after ruling out potential effects of age and sex. Specifically, a multiple linear regression model was utilized to rule out the effects of age and sex on morphological similarity between each pair of regions and each behavioral and cognitive variable. The resulting residuals were subsequently used to examine the relationship between morphological WM networks and behavioral and cognitive data via the PLS regression and to explore the ability of morphological WM networks to predict individual scores in behavioral and cognitive tests via the BBS modeling method. Notably, for the prediction analysis, all residuals were obtained from the multiple linear regression models constructed based on the training data.

### Effects of Image Quality

Accumulating evidence has highlighted the importance of controlling for the effects of in‐scanner head motion for studies of brain morphometry^[^ ^26^ ^]^ and morphological brain networks.^[^ ^27^ ^]^ However, there have been no effective methods to date to quantify head motion from a single T1‐weighted MRI image. To examine possible effects of head motion on the results, the IQRs generated by the CAT12 toolbox was used as a proxy for head motion since the amount of head motion during T1‐weighted MRI acquisition was previously demonstrated to be related to image quality of the acquired images.^[^ ^27^ ^]^ Specifically, the relationship (Spearman rank correlation) between the IQRs and interregional morphological similarity (HCP dataset, unrelated participants) was first examined using the TFNBS approach with the extension and height enhancement parameters set to 0.5 and 2.25, respectively.^[^ ^25^ ^]^ Then, the phenotypic correlates of morphological WM networks (HCP dataset, unrelated participants) were re‐analyzed after ruling out the effects of IQRs on morphological similarity of each edge and each behavioral and cognitive variable via a linear regression model. Notably, for the prediction analysis, all residuals were obtained from the linear regression models constructed based on the training data. Finally, the clinical value of morphological WM networks were re‐examined by treating individual IQRs as a covariate for all between‐group comparisons and by ruling out the effects of individual IQRs on morphological similarity (multiple linear regression) for all clinical correlation analyses.

### Statistical Analysis

The data were expressed as minimum–maximum for the age and *AR^2^* and mean ± standard deviation for the TRT reliability and heritability. The statistical methods used in this study included: 1) two‐tailed independent‐sample *T* test for the comparison of the morphological similarity between homotopic and heterotopic edges (*N* = 861 edges); 2) two‐way repeated ANOVA for the effects of different analytical strategies on the morphological similarity and TRT reliability of morphological WM networks (*N* = 1128 edges); 3) two‐tailed paired‐sample *T* test for post hoc comparisons of the effects of different analytical strategies on the morphological similarity and TRT reliability of morphological WM networks (*N* = 2256 edges for main effects, and *N* = 1128 edges for interaction effects), the differences in the heritability between the VBNs and DBNs (*N* = 1128 edges), and the effects of different smoothing kernel sizes on the TRT reliability (*N* = 1128 edges); 4) two‐tailed Spearman correlation for the relationship in the group‐level mean morphological similarity matrices (*N* = 1128 edges), the relationship between the heritability of edges and edges’ contributions to the phenotypic correlates of morphological WM networks (*N* = 1128 edges), the relationship between nodal *AR^2^* values and functional hierarchy, static metabolism, and neurotransmitter intensity (*N* = 48 regions), and the relationship between morphological similarity and clinical variables of the MS patients (*N* = 208 MS patients); 5) PLS regression for the relationship between morphological WM networks and behavioral and cognitive data (*N* = 444 unrelated participants in the HCP dataset) and the relationship between nodal *AR^2^* values and regional gene expression data (*N* = 38 regions); 6) BBS modeling method for the ability of morphological WM networks to predict individual scores in behavioral and cognitive tests (*N* = 444 unrelated participants in the HCP dataset); 6) communication model for the ability of morphological WM networks to predict the hamodynamic coherence, metabolic synchronization, structural connectivity, chemoarchitectonic covariance, and gene co‐expression networks (whole‐brain level: *N* = 703 edges for predicting gene co‐expression network; *N* = 1128 edges for predicting the other networks; nodal level: *N* = 37 edges for predicting gene co‐expression network; *N* = 47 edges for predicting the other networks); 7) TFNBS for the differences in interregional morphological similarity among the MS, NMOSD, and controls (*N* = 208 MS patients, 200 NMOSD patients, and 228 controls) and the relationship between interregional morphological similarity and IQRs (*N* = 444 unrelated participants in the HCP dataset). All statistical analyses were performed with MATLAB R2021b and *P* < 0.05 was considered statistically significant. Multiple comparisons were corrected if applicable.

## Conflict of Interest

The authors declare no conflict of interest.

## Supporting information

Supporting Information

## Data Availability

All data that support the findings of this study are from publicly available datasets (HCP dataset: www.humanconnectome.org; HNU dataset: https://doi.org/10.15387/fcp_indi.corr.hnu1; SWU dataset: https://doi.org/10.15387/fcp_indi.retro.slim; MU dataset: https://openneuro.org/datasets/ds002898/versions/1.1.0; JuSpace dataset: https://github.com/juryxy/JuSpace; AHBA dataset: http://human.brain‐map.org/) except for the MS and NMOSD multicentric dataset, which are available from the corresponding author upon reasonable request. Due to the nature of this research, participants of the MS and NMOSD multicentric dataset did not agree for their data to be shared publicly. Code for the main analyses excluding the neuroimaging preprocessing is available at https://github.com/Junle‐1995/Morphological‐WMNs.
