# Supplementary material for: Morphological Brain Networks of White Matter: Mapping, Evaluation, Characterization, and Application
Source: Adv Sci (Weinh). 2024 Jul 15;11(35):2400061. doi: 10.1002/advs.202400061 (PMC11425219; doi:10.1002/advs.202400061)
Supplement: Supplementary file 1 — Supporting Information [file ADVS-11-2400061-s001.docx]

**Supporting Information for**

**MORPHOLOGICAL BRAIN NETWORKS OF WHITE MATTER: MAPPING, EVALUATION, CHARACTERIZATION AND APPLICATION**

Junle Li, Suhui Jin, Zhen Li, Xiangli Zeng, Yuping Yang, Zhenzhen Luo, Xiaoyu Xu, Zaixu Cui, Yaou Liu, Jinhui Wang^*^

^*^Corresponding author: Jinhui Wang, PhD, Institute for Brain Research and Rehabilitation, South China Normal University, Guangzhou 510631, China. Tel: +86 20 8521 2189; Fax: +86 20 8521 2189; E-mail: jinhui.wang.1982@m.scnu.edu.cn

**
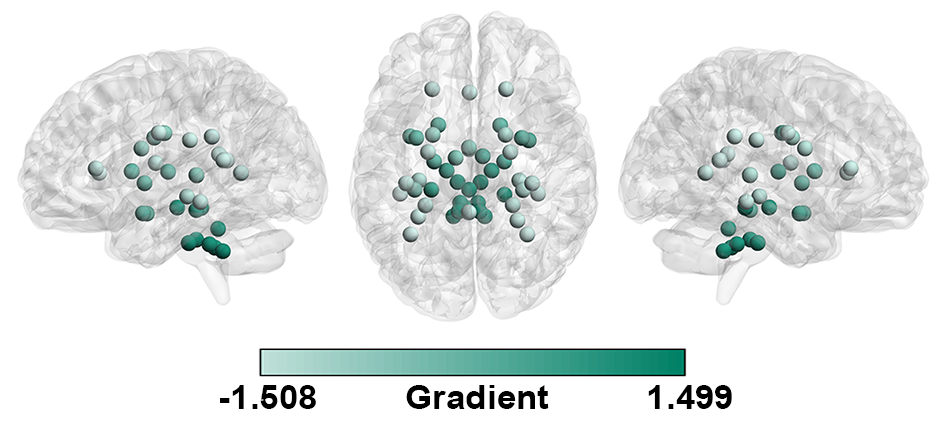
**

**Figure S1.** The first gradient derived from group-level mean hamodynamic coherence WM network. The first gradient accounted for 30.5% variance in the distribution of hamodynamic coherence across the WM regions, which was characterized by gradual reduction of hamodynamic coherence variance along an axis from inferior to superior white matter regions.

**
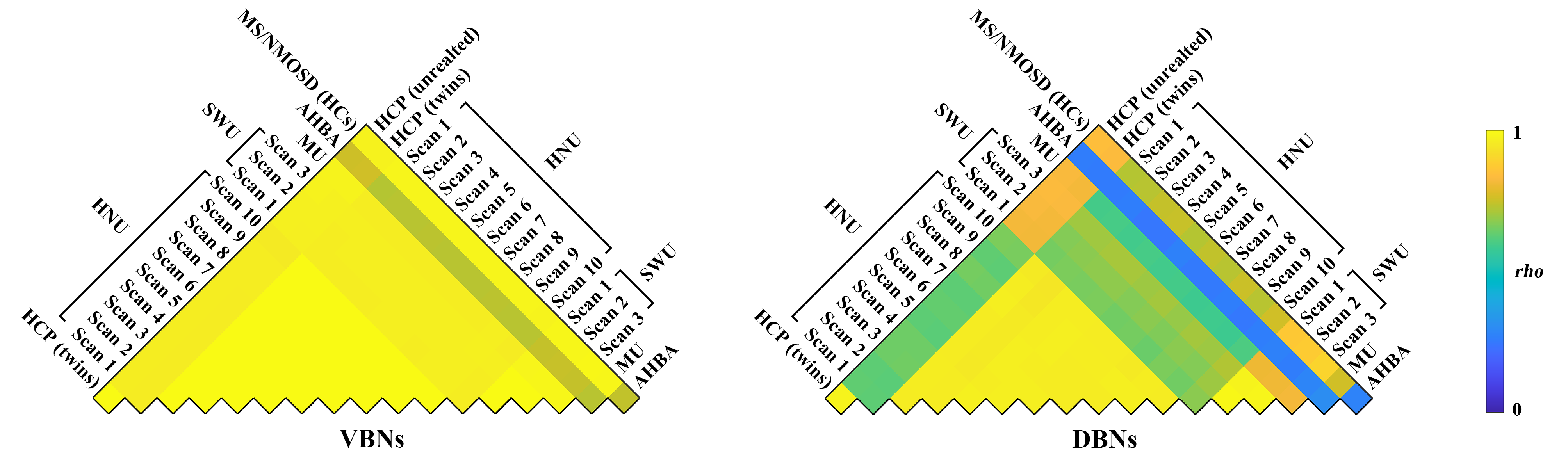
**

**Figure S2.** Cross-dataset similarity in the group-level mean similarity matrix of morphological WM networks. Significant correlations were observed between each pair of datasets (*N* = 1128 edges; Spearman rank correlation, *P* < 0.05, false discovery rate corrected). VBNs, volume-based networks; DBNs, deformation-based networks; HCP, Human Connectome Project; HNU, Hangzhou Normal University; SWU, South West University; MU, Monash University; AHBA, Allen Human Brain Atlas; MS, multiple sclerosis; NMOSD, neuromyelitis optica spectrum disorders; HCs, healthy controls.

**
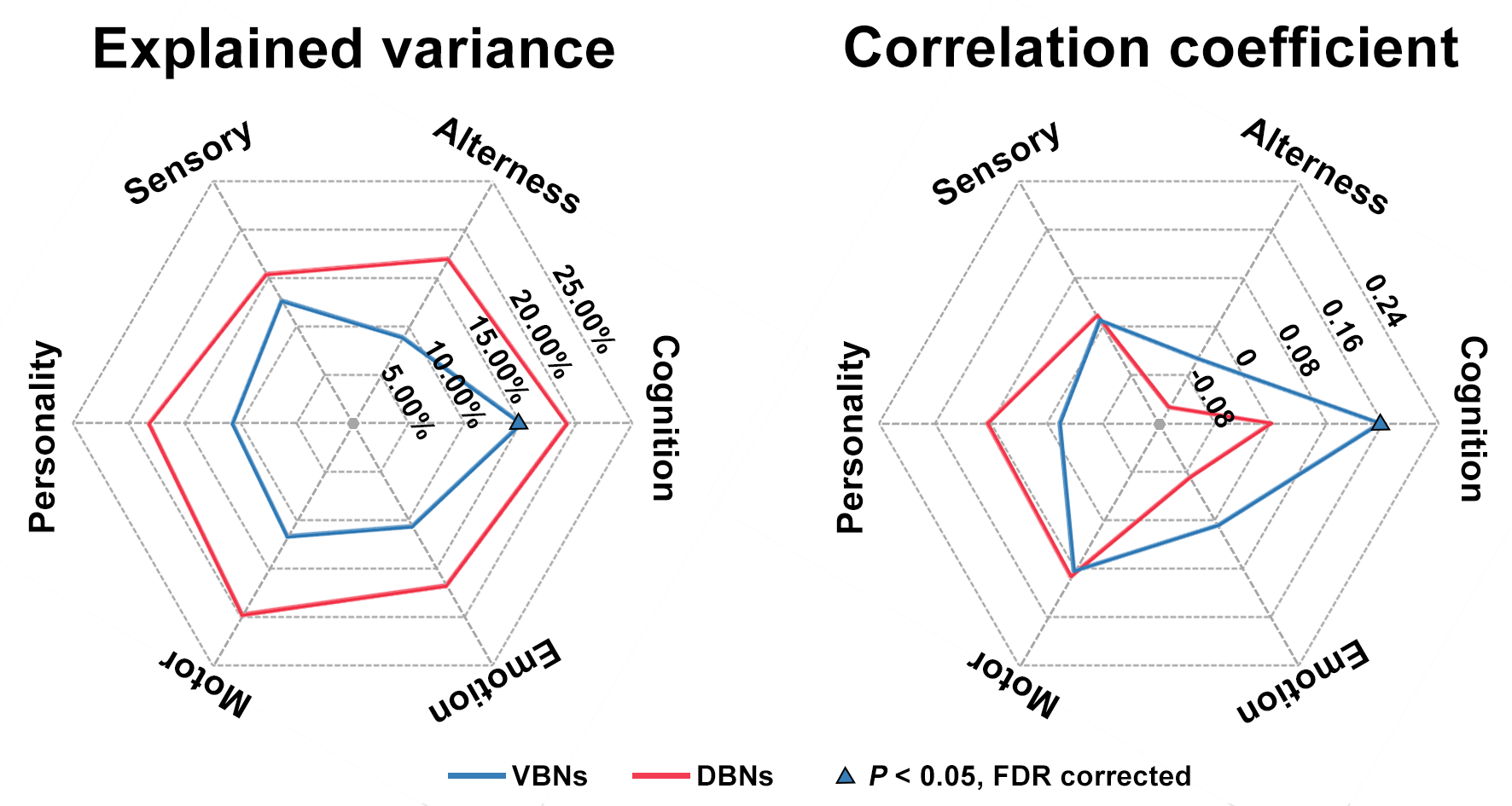
**

**Figure S3.** Behavioral and cognitive association of morphological WM networks after controlling for the effects of age and sex. The VBNs explained significant proportions of interindividual variance in the Cognition domain (PLS regression; left panel) and predicted individual scores of the Cognition domain (BBS modeling method; right panel) (*N* = 444 unrelated participants in the HCP dataset; *P* < 0.05, FDR corrected). VBNs, volume-based networks; DBNs, deformation-based networks; FDR, false discovery rate.

**
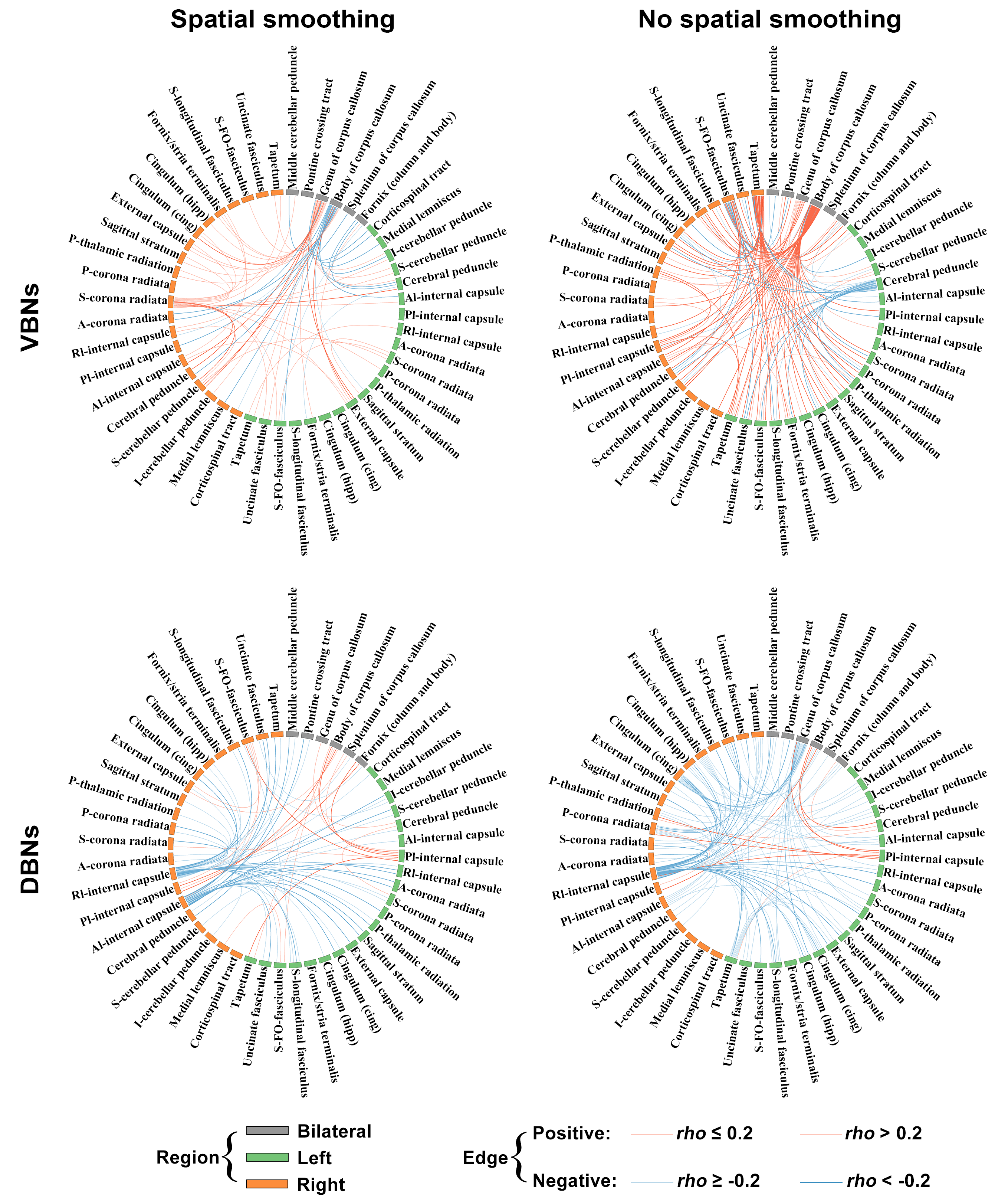
**

**Figure S4.** Edges showing significant correlations with the IQRs. A total of 76/111 edges in the VBNs and 69/126 edges in the DBNs were found to show significant correlations with the image quality of T1-weighted MRI images when morphological WM networks were constructed based on spatially smoothed/unsmoothed data (*N* = 444 unrelated participants in the HCP dataset; threshold-free network-based statistics approach, *P* < 0.05, family-wise error corrected). VBNs, volume-based networks; DBNs, deformation-based networks.

**
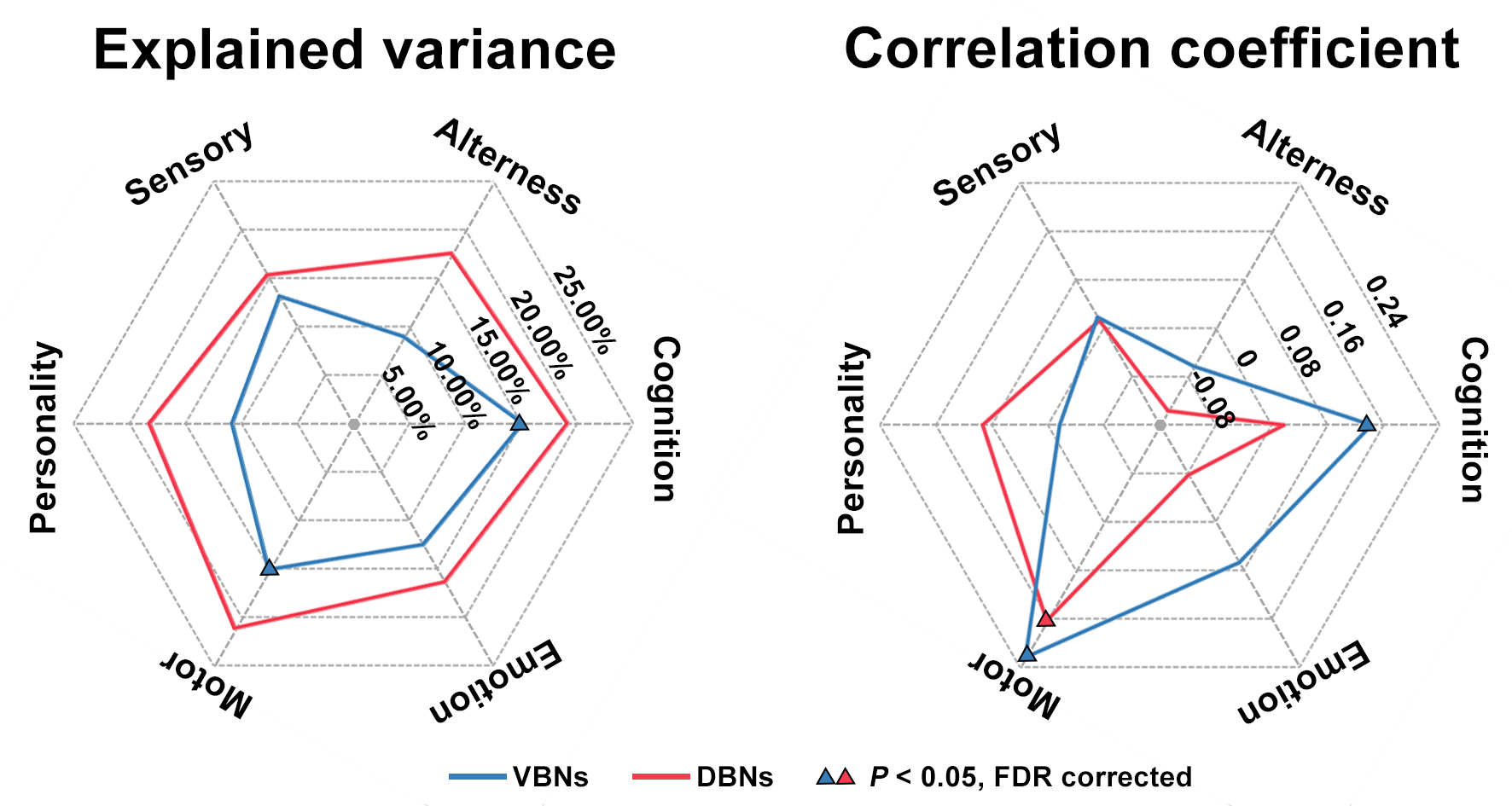
**

**Figure S5.** Behavioral and cognitive association of morphological WM networks after controlling for the effects of image quality. The VBNs explained significant proportions of interindividual variance in the Cognition and Motor domains (PLS regression; left panel) and the VBNs and DBNs significantly predicted individual scores of the Cognition and Motor domains and the Motor domain, respectively (BBS modeling method; right panel) (*N* = 444 unrelated participants in the HCP dataset; *P* < 0.05, FDR corrected). VBNs, volume-based networks; DBNs, deformation-based networks; FDR, false discovery rate.

**
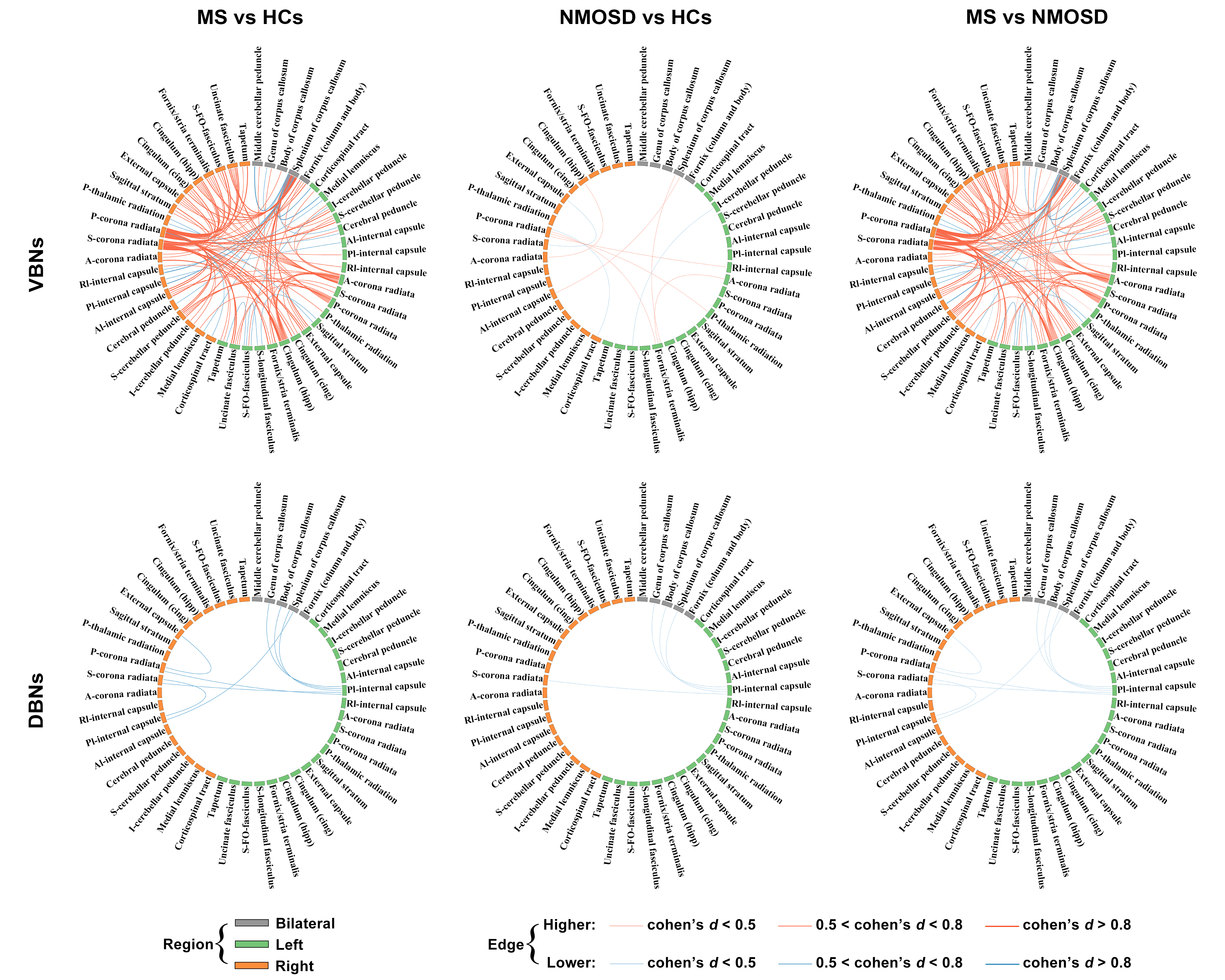
**

**Figure S6.** Edges showing significant between-group differences in the MS and NMOSD multicentric dataset after controlling for the effects of image quality. A total of 114/8 edges were identified to exhibit significant group main effects on interregional morphological similarities in the VBNs/DBNs (*N* = 208 MS patients, 200 NMOSD patients, and 228 HCs; threshold-free network-based statistics approach, *P* < 0.05, family-wise error corrected). VBNs, volume-based networks; DBNs, deformation-based networks; HCs, healthy controls; MS, multiple sclerosis; NMOSD, neuromyelitis optica spectrum disorders.

**
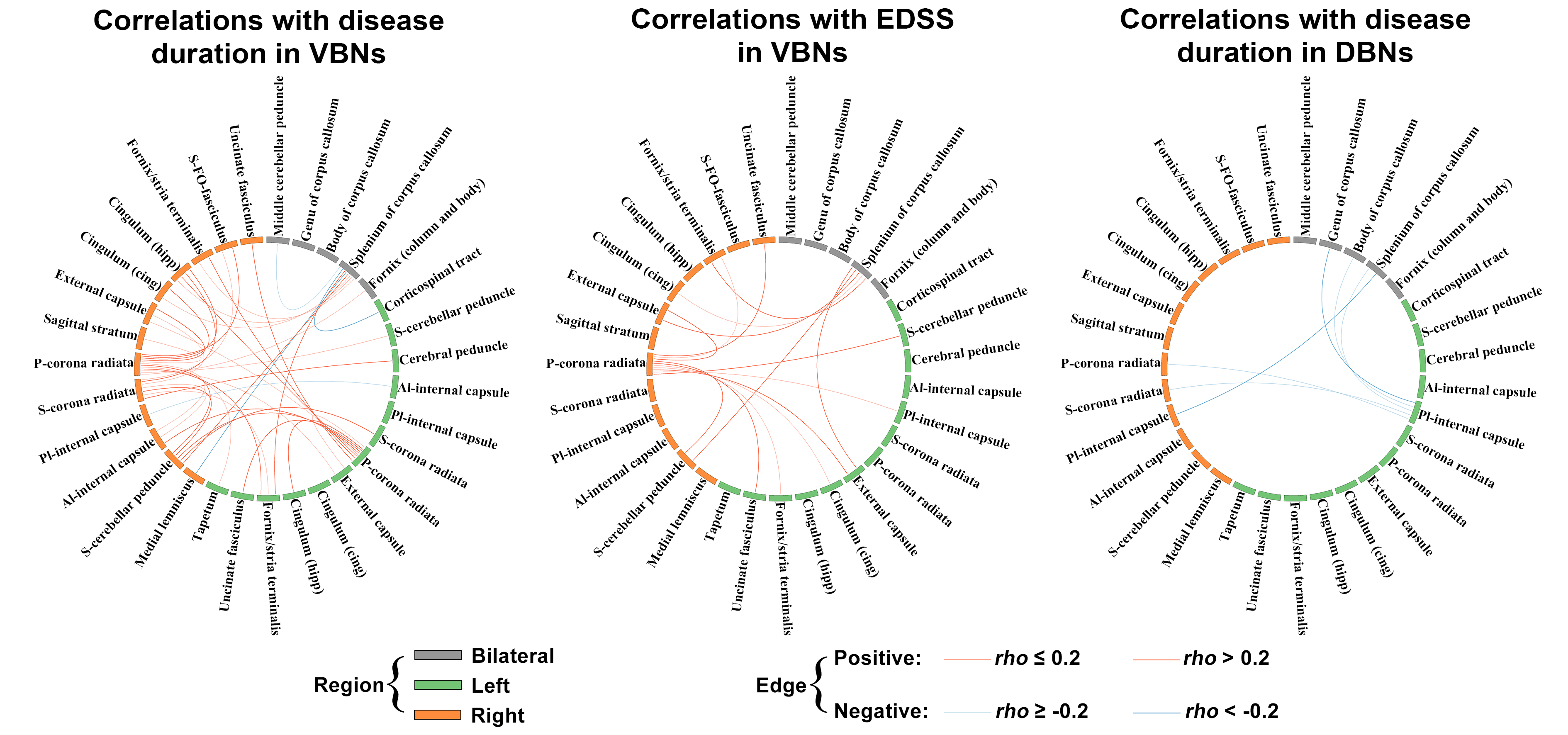
**

**Figure S7.** Edges showing significant correlations with clinical variables in the MS patients after controlling for the effects of image quality. For the VBNs, a total of 34/4 edges showed significant positive/negative correlations with disease duration (left panel) and a total of 15 edges showed significant positive correlations with EDSS scores (middle panel) of the MS patients (N = 208 MS patients; Spearman correlation, *P* < 0.05, false discovery rate corrected). For the DBNs, a total of 6 edges exhibited significant negative correlations with disease duration of the MS patients (N = 208 MS patients; Spearman correlation, *P* < 0.05, false discovery rate corrected; right panel). VBNs, volume-based networks; DBNs, deformation-based networks; EDSS, Expanded Disability Status Scale.

**Table S1**. Regions in the white matter atlas from Johns Hopkins University

| Index | Region | Hemisphere |
| --- | --- | --- |
| 1 | Middle cerebellar peduncle | B |
| 2 | Pontine crossing tract | B |
| 3 | Genu of corpus callosum | B |
| 4 | Body of corpus callosum | B |
| 5 | Splenium of corpus callosum | B |
| 6 | Fornix (column and body) | B |
| 7 | Corticospinal tract | L |
| 8 | Corticospinal tract | R |
| 9 | Medial lemniscus | L |
| 10 | Medial lemniscus | R |
| 11 | I-cerebellar peduncle | L |
| 12 | I-cerebellar peduncle | R |
| 13 | S-cerebellar peduncle | L |
| 14 | S-cerebellar peduncle | R |
| 15 | Cerebral peduncle | L |
| 16 | Cerebral peduncle | R |
| 17 | Al-internal capsule | L |
| 18 | Al-internal capsule | R |
| 19 | Pl-internal capsule | L |
| 20 | Pl-internal capsule | R |
| 21 | Rl-internal capsule | L |
| 22 | Rl-internal capsule | R |
| 23 | A-corona radiata | L |
| 24 | A-corona radiata | R |
| 25 | S-corona radiata | L |
| 26 | S-corona radiata | R |
| 27 | P-corona radiata | L |
| 28 | P-corona radiata | R |
| 29 | P-thalamic radiation | L |
| 30 | P-thalamic radiation | R |
| 31 | Sagittal stratum | L |
| 32 | Sagittal stratum | R |
| 33 | External capsule | L |
| 34 | External capsule | R |
| 35 | Cingulum (cing) | L |
| 36 | Cingulum (cingulate gyrus) | R |
| 37 | Cingulum (hipp) | L |
| 38 | Cingulum (hippocampus) | R |
| 39 | Fornix/stria terminalis | L |
| 40 | Fornix/stria terminalis | R |
| 41 | S-longitudinal fasciculus | L |
| 42 | S-longitudinal fasciculus | R |
| 43 | S-FO-fasciculus | L |
| 44 | S-FO-fasciculus | R |
| 45 | Uncinate fasciculus | L |
| 46 | Uncinate fasciculus | R |
| 47 | Tapetum | L |
| 48 | Tapetum | R |

A, anterior; Al, Anterior limb; FO, fronto-occipital; I, Inferior; P, posterior; S, superior; Pl, Posterior limb; Rl, Retrolenticular part; cing, cingulate gyrus; hipp, hippocampus; B, bilateral; L, left; R, right.

**Table S2**. Demographic variables of all participants in each dataset

| **Dataset** | **Sex (F/M)** | **Age^a)^ (years)** |
| --- | --- | --- |
| HCP (unrelated participants) | 239/205 | 96/202/141/5 |
| HCP (twin participants) | 260/174 | 54/212/164/4 |
| HNU | 15/15 | 24.367 (2.414) |
| SWU | 60/60 | 19.692 (0.942) |
| MU | 19/7 | 19.500 (1.364) |
| AHBA | 1/5 | 42.500 (13.383) |
| MS and NMOSD multicentric |  |  |
| HCs | 124/104 | 38.364 (12.089) |
| MS | 135/73 | 36.870 (11.339) |
| NMOSD | 175/25 | 41.135 (13.374) |

F, female; M, male; HCP, Human Connectome Project; HNU, Hangzhou Normal University; SWU, South West University; MU, Monash University; AHBA, Allen Human Brain Atlas; MS, multiple sclerosis; NMOSD, neuromyelitis optica spectrum disorders; HCs, health controls.

^a)^Data are presented as the number of participants within the age range of 22–25/26–30/31–35/>36 for the HCP dataset and mean (standard deviation) for the other datasets.

**Table S3.** Edges showing altered morphological similarities in MS and NMOSD and their correlations with clinical variables

| **Region** | **Region** | **Post hoc** | **MS**  **MS** | |
| --- | --- | --- | --- | --- |
|  |  |  | **Duration** | **EDSS** |
| **VBNs** |  |  |  |  |
| Middle cerebellar peduncle | Splenium of corpus callosum | HCs=NMOSD>MS | -0.205 |  |
| Genu of corpus callosum | Splenium of corpus callosum | HCs=NMOSD>MS |  |  |
| Splenium of corpus callosum | Fornix (column and body of fornix) | HCs=NMOSD<MS |  |  |
| Splenium of corpus callosum | L Corticospinal tract | HCs=NMOSD>MS | -0.260 |  |
| Splenium of corpus callosum | L Medial lemniscus | HCs=NMOSD>MS | -0.186 |  |
| Splenium of corpus callosum | R Medial lemniscus | HCs=NMOSD>MS | -0.232 |  |
| Splenium of corpus callosum | L Superior cerebellar peduncle | HCs=NMOSD<MS |  |  |
| Splenium of corpus callosum | R Superior cerebellar peduncle | HCs=NMOSD<MS | 0.291 | 0.221 |
| Splenium of corpus callosum | L Cerebral peduncle | HCs=NMOSD>MS |  |  |
| Splenium of corpus callosum | R Cerebral peduncle | HCs=NMOSD>MS |  |  |
| Splenium of corpus callosum | R Anterior limb of internal capsule | HCs<NMOSD<MS |  |  |
| Splenium of corpus callosum | R Posterior limb of internal capsule | HCs=NMOSD>MS |  |  |
| L Anterior limb of internal capsule | R Posterior limb of internal capsule | HCs=NMOSD>MS | -0.219 |  |
| Splenium of corpus callosum | R Retrolenticular part of internal capsule | HCs=NMOSD>MS |  |  |
| L Medial lemniscus | R Retrolenticular part of internal capsule | HCs=NMOSD>MS |  |  |
| Fornix (column and body of fornix) | L Anterior corona radiata | HCs=NMOSD<MS |  |  |
| L Inferior cerebellar peduncle | L Anterior corona radiata | HCs=NMOSD<MS |  |  |
| L Superior cerebellar peduncle | L Anterior corona radiata | HCs=NMOSD<MS |  |  |
| R Superior cerebellar peduncle | L Anterior corona radiata | HCs=NMOSD<MS |  |  |
| R Anterior limb of internal capsule | L Anterior corona radiata | HCs=NMOSD<MS |  |  |
| L Retrolenticular part of internal capsule | L Anterior corona radiata | HCs<NMOSD<MS |  |  |
| R Superior cerebellar peduncle | L Superior corona radiata | HCs=NMOSD<MS | 0.226 |  |
| Fornix (column and body of fornix) | R Superior corona radiata | HCs=NMOSD<MS |  |  |
| L Inferior cerebellar peduncle | R Superior corona radiata | HCs=NMOSD<MS |  |  |
| R Inferior cerebellar peduncle | R Superior corona radiata | HCs=NMOSD<MS |  |  |
| L Superior cerebellar peduncle | R Superior corona radiata | HCs=NMOSD<MS | 0.182 |  |
| R Superior cerebellar peduncle | R Superior corona radiata | HCs=NMOSD<MS | 0.247 |  |
| L Cerebral peduncle | R Superior corona radiata | HCs=NMOSD<MS | 0.250 |  |
| R Anterior limb of internal capsule | R Superior corona radiata | HCs=NMOSD<MS | 0.204 |  |
| R Superior cerebellar peduncle | L Posterior corona radiata | HCs=NMOSD<MS | 0.271 |  |
| R Anterior limb of internal capsule | L Posterior corona radiata | HCs=NMOSD<MS | 0.244 |  |
| Body of corpus callosum | R Posterior corona radiata | HCs=NMOSD<MS |  |  |
| Fornix (column and body of fornix) | R Posterior corona radiata | HCs=NMOSD<MS | 0.208 |  |
| R Corticospinal tract | R Posterior corona radiata | HCs>NMOSD>MS |  |  |
| L Inferior cerebellar peduncle | R Posterior corona radiata | HCs=NMOSD<MS |  |  |
| R Inferior cerebellar peduncle | R Posterior corona radiata | HCs=NMOSD<MS | 0.174 |  |
| L Superior cerebellar peduncle | R Posterior corona radiata | HCs=NMOSD<MS | 0.197 | 0.227 |
| R Superior cerebellar peduncle | R Posterior corona radiata | HCs=NMOSD<MS | 0.285 | 0.236 |
| R Anterior limb of internal capsule | R Posterior corona radiata | HCs=NMOSD<MS | 0.218 |  |
| L Posterior limb of internal capsule | R Posterior corona radiata | HCs=NMOSD<MS |  | 0.181 |
| L Retrolenticular part of internal capsule | R Posterior corona radiata | HCs<NMOSD<MS |  |  |
| R Posterior corona radiata | L Posterior thalamic radiation | HCs<NMOSD<MS | 0.176 |  |
| L Cerebral peduncle | R Posterior thalamic radiation | HCs=NMOSD>MS |  |  |
| R Posterior thalamic radiation | L Sagittal stratum | HCs=NMOSD>MS |  |  |
| L Anterior corona radiata | R Sagittal stratum | HCs=NMOSD<MS |  |  |
| R Superior corona radiata | R Sagittal stratum | HCs=NMOSD<MS |  |  |
| R Posterior corona radiata | R Sagittal stratum | HCs=NMOSD<MS | 0.208 |  |
| Splenium of corpus callosum | L External capsule | HCs=NMOSD<MS |  | 0.237 |
| L Anterior corona radiata | L External capsule | HCs=NMOSD<MS |  |  |
| R Superior corona radiata | L External capsule | HCs=NMOSD<MS | 0.174 |  |
| L Posterior corona radiata | L External capsule | HCs=NMOSD<MS | 0.209 | 0.173 |
| R Posterior corona radiata | L External capsule | HCs=NMOSD<MS | 0.214 | 0.235 |
| Splenium of corpus callosum | R External capsule | HCs=NMOSD<MS | 0.193 | 0.214 |
| L Anterior corona radiata | R External capsule | HCs=NMOSD<MS |  |  |
| R Superior corona radiata | R External capsule | NMOSD<HCs<MS | 0.217 |  |
| L Posterior corona radiata | R External capsule | HCs=NMOSD<MS | 0.223 |  |
| R Posterior corona radiata | R External capsule | HCs=NMOSD<MS | 0.240 | 0.210 |
| L Anterior corona radiata | L Cingulum (cingulate gyrus) | HCs=NMOSD<MS |  |  |
| R Superior corona radiata | L Cingulum (cingulate gyrus) | HCs=NMOSD<MS |  |  |
| R Posterior corona radiata | L Cingulum (cingulate gyrus) | HCs=NMOSD<MS |  | 0.191 |
| Splenium of corpus callosum | R Cingulum (cingulate gyrus) | HCs=NMOSD<MS | 0.208 | 0.182 |
| L Anterior corona radiata | R Cingulum (cingulate gyrus) | HCs=NMOSD<MS |  |  |
| R Superior corona radiata | R Cingulum (cingulate gyrus) | HCs=NMOSD<MS | 0.197 |  |
| R Posterior corona radiata | R Cingulum (cingulate gyrus) | HCs=NMOSD<MS | 0.228 |  |
| Middle cerebellar peduncle | L Cingulum (hippocampus) | HCs=NMOSD<MS |  |  |
| Genu of corpus callosum | L Cingulum (hippocampus) | HCs=NMOSD<MS |  |  |
| Splenium of corpus callosum | L Cingulum (hippocampus) | HCs=NMOSD<MS |  |  |
| L Corticospinal tract | L Cingulum (hippocampus) | HCs=NMOSD<MS |  |  |
| R Medial lemniscus | L Cingulum (hippocampus) | HCs=NMOSD<MS |  |  |
| R Retrolenticular part of internal capsule | L Cingulum (hippocampus) | HCs=NMOSD<MS |  |  |
| L Anterior corona radiata | L Cingulum (hippocampus) | HCs=NMOSD<MS |  |  |
| R Anterior corona radiata | L Cingulum (hippocampus) | HCs=NMOSD<MS |  |  |
| L Superior corona radiata | L Cingulum (hippocampus) | HCs=NMOSD<MS |  |  |
| R Superior corona radiata | L Cingulum (hippocampus) | HCs=NMOSD<MS | 0.170 |  |
| L Posterior corona radiata | L Cingulum (hippocampus) | HCs=NMOSD<MS | 0.232 |  |
| R Posterior corona radiata | L Cingulum (hippocampus) | HCs=NMOSD<MS | 0.172 |  |
| Splenium of corpus callosum | R Cingulum (hippocampus) | HCs=NMOSD<MS | 0.216 |  |
| L Anterior corona radiata | R Cingulum (hippocampus) | HCs=NMOSD<MS |  |  |
| R Anterior corona radiata | R Cingulum (hippocampus) | HCs<NMOSD<MS |  |  |
| R Superior corona radiata | R Cingulum (hippocampus) | HCs=NMOSD<MS | 0.221 |  |
| L Posterior corona radiata | R Cingulum (hippocampus) | HCs=NMOSD<MS | 0.286 |  |
| R Posterior corona radiata | R Cingulum (hippocampus) | HCs=NMOSD<MS | 0.252 |  |
| Splenium of corpus callosum | L Fornix (cres) / Stria terminalis | HCs<NMOSD<MS | 0.249 |  |
| L Anterior corona radiata | L Fornix (cres) / Stria terminalis | HCs<NMOSD<MS |  |  |
| R Superior corona radiata | L Fornix (cres) / Stria terminalis | HCs=NMOSD<MS | 0.197 |  |
| R Posterior corona radiata | L Fornix (cres) / Stria terminalis | HCs<NMOSD<MS | 0.262 | 0.194 |
| Splenium of corpus callosum | R Fornix (cres) / Stria terminalis | HCs=NMOSD<MS | 0.196 | 0.220 |
| L Anterior corona radiata | R Fornix (cres) / Stria terminalis | HCs=NMOSD<MS |  |  |
| R Superior corona radiata | R Fornix (cres) / Stria terminalis | HCs=NMOSD<MS |  |  |
| L Posterior corona radiata | R Fornix (cres) / Stria terminalis | HCs=NMOSD<MS | 0.238 |  |
| R Posterior corona radiata | R Fornix (cres) / Stria terminalis | HCs=NMOSD<MS | 0.189 | 0.199 |
| L Sagittal stratum | L Superior longitudinal fasciculus | HCs=NMOSD>MS |  |  |
| L Inferior cerebellar peduncle | L Superior fronto-occipital fasciculus | HCs>NMOSD>MS |  |  |
| L Cingulum (hippocampus) | L Superior fronto-occipital fasciculus | HCs=NMOSD<MS |  |  |
| L Superior longitudinal fasciculus | L Superior fronto-occipital fasciculus | HCs=NMOSD>MS |  |  |
| Splenium of corpus callosum | R Superior fronto-occipital fasciculus | HCs=NMOSD<MS | 0.180 | 0.170 |
| L Anterior corona radiata | R Superior fronto-occipital fasciculus | HCs=NMOSD<MS |  |  |
| R Superior corona radiata | R Superior fronto-occipital fasciculus | HCs=NMOSD<MS | 0.204 |  |
| R Posterior corona radiata | R Superior fronto-occipital fasciculus | HCs=NMOSD<MS | 0.234 |  |
| Splenium of corpus callosum | L Uncinate fasciculus | HCs=NMOSD<MS |  |  |
| L Anterior corona radiata | L Uncinate fasciculus | HCs=NMOSD<MS |  |  |
| R Superior corona radiata | L Uncinate fasciculus | HCs=NMOSD<MS | 0.171 |  |
| L Posterior corona radiata | L Uncinate fasciculus | HCs=NMOSD<MS | 0.224 |  |
| R Posterior corona radiata | L Uncinate fasciculus | HCs=NMOSD<MS | 0.193 | 0.217 |
| Splenium of corpus callosum | R Uncinate fasciculus | HCs=NMOSD<MS |  |  |
| R Medial lemniscus | R Uncinate fasciculus | HCs=NMOSD<MS |  |  |
| L Anterior corona radiata | R Uncinate fasciculus | HCs=NMOSD<MS |  |  |
| R Anterior corona radiata | R Uncinate fasciculus | HCs=NMOSD<MS |  | 0.170 |
| R Superior corona radiata | R Uncinate fasciculus | HCs=NMOSD<MS |  |  |
| L Posterior corona radiata | R Uncinate fasciculus | HCs=NMOSD<MS | 0.223 |  |
| R Posterior corona radiata | R Uncinate fasciculus | HCs=NMOSD<MS | 0.171 | 0.230 |
| R Superior corona radiata | L Tapetum | HCs=NMOSD<MS | 0.173 |  |
| R Posterior corona radiata | L Tapetum | HCs=NMOSD<MS | 0.217 |  |
| L Superior fronto-occipital fasciculus | L Tapetum | HCs=NMOSD>MS |  |  |
| R Superior corona radiata | R Tapetum | HCs=NMOSD<MS |  |  |
| R Posterior corona radiata | R Tapetum | HCs=NMOSD<MS |  | 0.169 |
| **DBNs** |  |  |  |  |
| Genu of corpus callosum | L Posterior limb of internal capsule | HCs>NMOSD>MS | -0.222 |  |
| Body of corpus callosum | L Posterior limb of internal capsule | HCs>NMOSD>MS |  |  |
| Splenium of corpus callosum | L Posterior limb of internal capsule | HCs>NMOSD>MS | -0.186 |  |
| Splenium of corpus callosum | R Posterior limb of internal capsule | HCs=NMOSD>MS | -0.229 |  |
| L Posterior limb of internal capsule | R Superior corona radiata | HCs>NMOSD>MS |  |  |
| R Posterior limb of internal capsule | R Superior corona radiata | HCs=NMOSD>MS |  |  |
| L Posterior limb of internal capsule | R Posterior corona radiata | HCs=NMOSD>MS |  |  |
| R Posterior corona radiata | R External capsule | HCs=NMOSD>MS |  |  |

Only significant Spearman rank correlations are shown. DBNs, deformation-based networks; VBNs, volume-based networks; HCs, healthy controls; MS, multiple sclerosis; NMOSD, neuromyelitis optica spectrum disorders; L, left; R, right; EDSS, Expanded Disability Status Scale.

**Table S4**. Test-retest reliability of morphological similarity when different smoothing kernel sizes are used

|  | **Smoothing kernel size** | | | | |
| --- | --- | --- | --- | --- | --- |
|  | 2-mm | 4-mm | 6-mm | | 8-mm |
| **Short-term** | | | |  |  |
| VBNs | 0.781 (0.148) | 0.795 (0.145) | 0.809 (0.134) | | 0.832 (0.115) |
| DBNs | 0.757 (0.132) | 0.774 (0.120) | 0.783 (0.117) | | 0.803 (0.104) |
| **Long-term** | | | |  |  |
| VBNs | 0.797 (0.129) | 0.813 (0.123) | 0.833 (0.106) | | 0.844 (0.103) |
| DBNs | 0.776 (0.114) | 0.789 (0.107) | 0.800 (0.097) | | 0.816 (0.086) |

Data are presented as mean (standard deviation). VBNs, volume-based networks; DBNs, deformation-based networks.

**Table S5**. Frechet distance between probability density functions estimated with different numbers of sampling points

|  | **Spatial smoothing** | **No spatial smoothing** |
| --- | --- | --- |
| **Volume** | | |
| 2^4^ vs 2^5^ | 0.089 (0.015) | 0.128 (0.038) |
| 2^5^ vs 2^6^ | 0.043 (0.008) | 0.067 (0.024) |
| 2^6^ vs 2^7^ | 0.021 (0.004) | 0.034 (0.013) |
| 2^7^ vs 2^8^ | 0.011 (0.002) | 0.017 (0.006) |
| 2^8^ vs 2^9^ | 0.005 (0.001) | 0.008 (0.003) |
| 2^9^ vs 2^10^ | 0.003 (0.000) | 0.004 (0.002) |
| **Deformation** | | |
| 2^4^ vs 2^5^ | 0.093 (0.008) | 0.105 (0.013) |
| 2^5^ vs 2^6^ | 0.045 (0.004) | 0.052 (0.008) |
| 2^6^ vs 2^7^ | 0.022 (0.002) | 0.026 (0.004) |
| 2^7^ vs 2^8^ | 0.011 (0.001) | 0.013 (0.002) |
| 2^8^ vs 2^9^ | 0.006 (0.001) | 0.006 (0.001) |
| 2^9^ vs 2^10^ | 0.003 (0.000) | 0.003 (0.000) |

Data are represented as mean (standard deviation).
